# The Fungal Microbiome Associated with *Schizaphis graminum* Biotypes E and K in Response to Host Plant Species, Cultivar, and Elapsed Time

**DOI:** 10.3390/insects17101025

**Published:** 2026-10-05

**Authors:** Yan M. Crane, Charles F. Crane, Craig D. Tipton, Jacob S. Ancira, Brandon J. Schemerhorn

**Affiliations:** 1 Crop Production and Pest Control Research Unit, USDA-ARS, West Lafayette, IN 47907, USA; 2 Department of Entomology, Purdue University, West Lafayette, IN 47907, USA; 3 Department of Botany and Plant Pathology, Purdue University, West Lafayette, IN 47907, USA; 4 RTL Genomics, MicroGenDX, Lubbock, TX 79413, USA

**Keywords:** *Schizaphis graminum*, microbiome, 18S rRNA, infestation duration, dietary influence

## Abstract

Because so little is known about fungal microbiomes associated with aphids, the fungal mycobiota associated with feeding by biotypes E and K of greenbug aphid (*Schizaphis graminum*) was surveyed using ITS amplicon sequences with regard to host plant species, days post infestation, host plant cultivar, and host plant susceptibility to greenbug feeding. The host plant species were wheat (*Triticum aestivum* L.), rye (*Secale cereale* L.), barley (*Hordeum vulgare* L.), sorghum (*Sorghum bicolor* (L.) Moench), and *Aegilops triuncialis* L., and there were 17 cultivars or accessions among these species. Biotype, collection date, host plant species, host plant cultivar, and host plant susceptibility ranked strongest to weakest in influence on fungal community composition, and all influences were statistically significant except susceptibility, but biotype had by far the greatest influence. Filamentous ascomycetes and yeast-like basidiomycetes dominated the diverse fungal microbiota, and many reads mapped to unidentified taxa at the phylum or class level. Prominent genera of filamentous fungi included *Fusarium*, *Mortierella*, *Simplicillium*, *Arthrobotrys*, and “Capnodiales genus incertae sedis”. Prominent yeast genera included *Hannaella*, *Papiliotrema*, *Naganishia*, and *Rhodotorula* among basidiomycetes and “Debaryomycetaceae genus incertae sedis” among ascomycetes. Sister samples of each combination of biotype, cultivar, and collection date could differ greatly in frequency of dominant genera, and individual genera frequently bloomed to high frequency with biotype K. Community diversity decreased at the end of the time course. Analysis of frequency correlations with weighted gene co-expression network analysis (WGCNA) identified six modules of 10 to 28 correlated genera in biotype K and five modules in biotype E. At least five genera in a “*Hannaella*” module have documented antifungal activity. The sample collection failed to separate greenbugs from adhering honeydew or fragments of the leaf where they were feeding, and therefore the location of the detected fungi is not known, which limits the inferences that can be drawn from correlations of taxon frequencies. Most importantly, there is still no proof that a gut microbiota exists in greenbug. Nevertheless, numerous negative correlations in taxon frequency implied that more than one fungal community was present. Further investigation with microscopy is needed to locate fungi in or on the greenbug or at its feeding site.

## Simple Summary

The greenbug aphid is a major pest of wheat and sorghum. It depends on a special bacterium to survive, and this bacterium is well understood. Nothing is known about fungi that grow and interact with greenbug. Therefore, we used DNA sequences to census fungi from greenbug and seek hints about their interaction with greenbug strain, host plant, time, and one another. We identified 480 kinds of fungi and found that the strain of greenbug most influenced which fungi were most common. Some of these fungi became less common over time and were replaced by other fungi. Many of these fungi were yeasts adapted to life in sugary solutions like the honeydew that greenbugs produce. Because the aphid mouth appears to be too small to swallow fungal cells, these fungi were probably in honeydew that surrounded and adhered to the greenbug. Further experiments are needed to precisely locate these fungi in, on, or near greenbugs. Our findings add to basic knowledge about aphid interactions with fungi, and this basic knowledge might eventually be useful in controlling greenbugs on cereal crops.

## 1. Introduction

The greenbug aphid, *Schizaphis graminum* (Rondani), is a sap-sucking insect that has been observed to feed on at least 70 species of grasses [1]. It has been an especially serious pest on wheat and sorghum. Annual economic loss to greenbug and its vectored yellow-dwarf disease in the United States during the 1990s outbreaks exceeded 100 million then-current dollars [2]. Greenbug damages wheat through direct feeding and transmission of yellow-dwarf viruses. Greenbug saliva is toxic to wheat and causes yellow to reddish spots at the feeding sites. Heavy infestation can lead to chlorosis and plant death even in the absence of yellow-dwarf disease [3,4]. Multiple strains of greenbug exist in the United States. These strains are termed biotypes; they are defined by their virulence against different varieties of the host plant that carry different resistance genes [3,5] and not by genetic uniformity or common ancestry. Biotype E was first reported in 1979 on wheat [1] and apparently remains the prevalent biotype in the United States at large. Biotype K was first reported on sorghum in 1992 [1] and has mostly replaced biotype E in Kansas, where biotype K infests deployed sorghum hybrids that resist biotype E.

In aphids, bacterial microbiomes have received considerable attention because of the presence of an intracellular endosymbiont, *Buchnera aphidicola*, which improves the nutritional balance of amino acids in their diet of plant sap. Various studies have documented additional bacterial symbionts that can partially substitute for *B. aphidicola* and provide B-vitamins [6]. Fungal microbiomes have attracted far less attention in aphids. Initially, Fukatsu and Ishikawa [7] identified a potentially mutualistic yeast in *Hamiltonaphis styraci* as a pyrenomycete and deposited an 18S rDNA sequence (NCBI accession D55719.1) from it at GenBank. Blastn of this sequence against UNITE [8] hit most closely a sordariomycete, *Cordyceps fumosorosea*, a known insect pathogen, at 96% identity. Grigorescu et al. [9] cultured six fungal strains from 15 aphid strains in seven aphid species. They identified four strains of *Cryptococcus* and one each of *Debaryomyces* and *Symmetrospora* on the basis of 18S rRNA gene sequences. Yang et al. [10] found 97 OTUs in 33 genera in amplified ITS sequences from *Melanaphis bambusae* that were healthy and recently fed, healthy but starved, or infected with *Conidiobolus obscurus.* Ascomycota comprised more than 90% of the fungal reads, and class Saccharomycetes accounted for about half of the fungal reads. Genera *Alternaria*, *Calyptella*, *Bipolaris*, *Cladophialophora*, *Phaeosphaeriopsis*, *Pichia*, and *Sporobolomyces* were overrepresented in healthy aphids. After removal of *Conidiobolus* reads, the Chao1 richness index ranged from 63.14 to 66.12 across the three groups, but the Shannon entropy was much lower in starved (0.66) than in healthy (2.11) or infected (2.29) aphids. There was a large increase in three *Cladosporium* species and an equivalent decrease in *Pichia guilliermondii* in the starved group. The Simpson index likewise was low in starved aphids (0.16) versus the healthy (0.67) or infected (0.64) groups. Besides *Conidiobolus obscurus*, several fungi commonly cause disease in aphids, including *Pandora neoaphidis*, *Entomophthora planchoniana*, and *Neozygites fresenii* [11].

Wolfgang et al. [12] investigated bacterial and fungal microbiomes in the common oak aphid, *Tuberculatus annulatus*, with 16S rRNA and ITS sequences in relation to foliar and soil microbiota on host oak seedlings. Three different soil microbiota differentially promoted species of *Mortierella*, *Parasola*, *Cladosporium*, unidentified Lasiosphaeriaceae, unidentified Pleosporales, unidentified Helotiales, and unidentified Agaricomycetes in the aphids. Species richness, Shannon entropy, and abundance did not vary significantly among the treatments.

In other Hemiptera, Gonzalez-Roman et al. [13] identified eight genera of fungi from the gut, ovary, fat body, and hemolymph in visibly healthy *Dactylopius coccus*, the cochineal scale insect. They provided micrographs of fungal hyphae in these body sites. They showed that these genera collectively produced cellulase, pectinase, and amylase enzymes that could degrade complex polysaccharides that are ingested with the rest of the sap from the host prickly pear cactus, thereby increasing caloric intake. These enzymatic activities decreased when the scale insects fed on cactus that had been treated with fungicides.

Crane et al. [14,15] surveyed bacteria and some fungi in four greenbug biotypes in two ways and obtained strikingly contrasting results. By mapping mRNA reads from an RNA-seq project against some 63,000 whole genomes of bacteria, archaea, viruses, fungi, and protozoa, Crane et al. [14] found 253 fungal genera. The ten most abundant genera in order of decreasing abundance were *Gilbertella*, *Malassezia*, *Mucor*, *Ascochyta*, *Protomyces*, *Mycotypha*, *Parastagonospora*, *Moesziomyces*, *Fusarium*, and *Dichomitus*. Fungi were rarer than bacteria and accounted for only 3.3% of all microbial reads. The contrast with 16S amplicon censusing [15] involved bacteria. With mRNA from biotypes B and H, enteric genera including *Shigella*, *Escherichia*, and *Desulfovibrio* dominated the microbiota, and the endosymbiotic *Buchnera* ranked behind *Shigella* and *Desulfovibrio*. With 16S rRNA amplicons from biotypes E and K, *Desulfovibrio* was not detected at all, even though there were more than 500 *Desulfovibrio* sequences in the Silva database [16]. Instead, *Buchnera* overwhelmingly outnumbered all other genera and comprised 78.24 to 99.99% of the read counts among samples. *Buchnera*’s increased 16S dominance was attributed to its high copy number of genomes per cell [17]. Correlation among abundances revealed two groups of coordinately populated taxa [15]. One group consisted of *Citrobacter koseri*, *Escherichia coli*, *Pantoea dispersa*, *Chryseobacterium cheonjiense*, and an unidentified species of *Enterobacter*. The other group consisted of 16 less-enteric taxa that consistently increased when *Buchnera* decreased.

Our current study involved fungi in the same greenbug DNA samples surveyed for bacteria by Crane et al. [15]. The internal transcribed spacer region (ITS) was amplified from the loci that encode 18S rRNA. Sequence counts were collated by taxon to assess fungal community composition and diversity. The experiment was designed under the hypotheses that greenbugs have a fungal microbiota; that this microbiota responds to greenbug genotype, host plant, time since infestation, and host plant resistance to greenbug feeding; and that ITS sequencing and RNA-seq detect the same fungal taxa. The experiment was not designed to locate fungi in the aphid samples and therefore lacked aphid-free controls or microscopic documentation, which in retrospect was a serious flaw that limits what can be inferred from taxon frequencies. It is possible to propose mechanistic hypotheses from frequency correlations, but these hypotheses must be addressed in their own future experiments. We will discuss three such hypotheses: (1) that greenbug placement on a new plant starts an ecological succession as honeydew is introduced to the leaf surface and ages there; (2) that a honeydew microbiota is perpetuated across aphid generations as aphids touch old honeydew and then touch and contaminate new honeydew; and (3) that negative correlations in taxon frequency reflect the existence of discrete communities in or on or surrounding the greenbugs, and that spatial separation or antagonistic interactions help to maintain these communities.

## 2. Materials and Methods

### 2.1. Sample Sources and Collection

**Figure 1:**
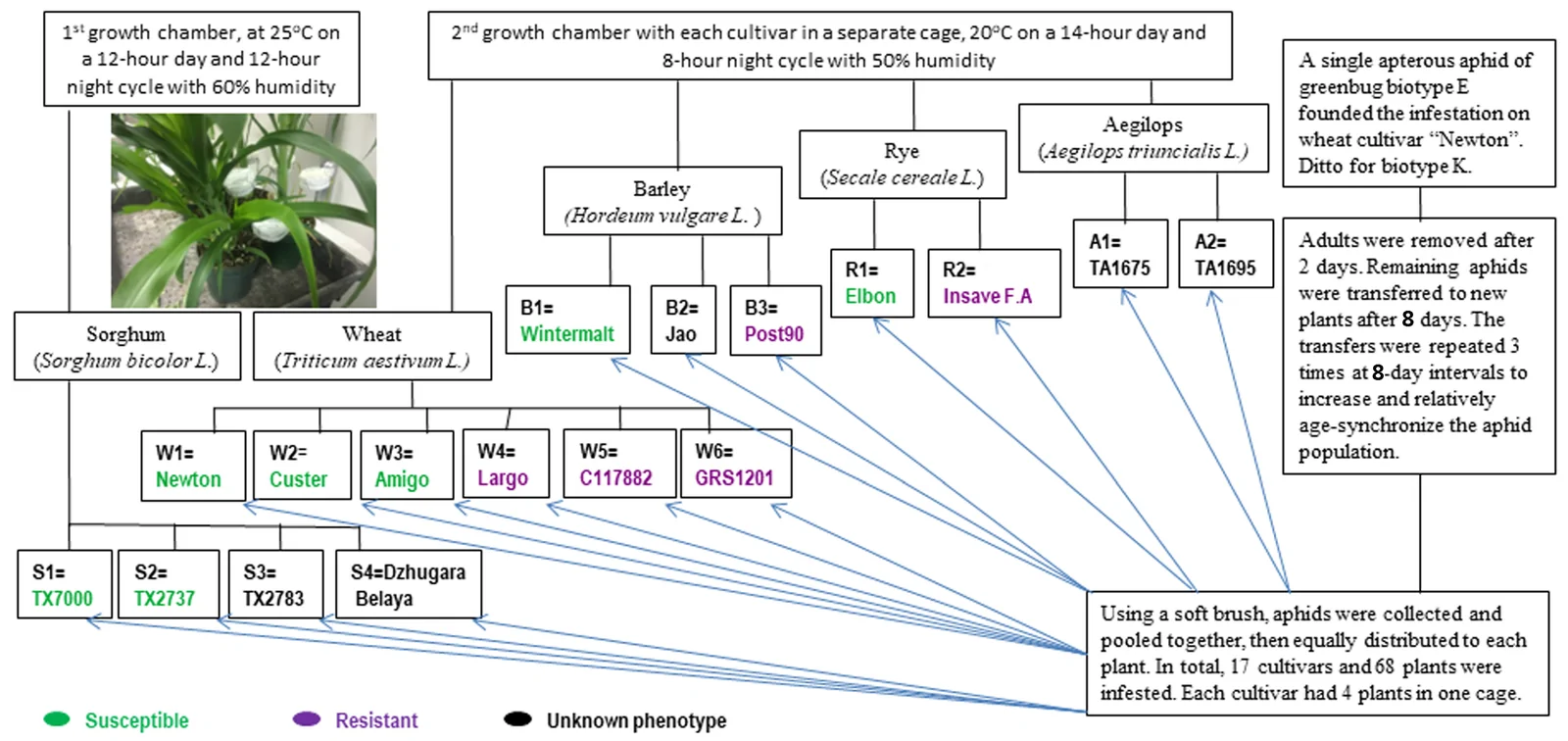
Experimental layout. Arrows indicate distribution from a pool of aphids to the different cultivars.

Greenbug biotypes E and K were used. Greenbug source, population increase, and infestation proceeded exactly as described in our sister study with the V5-V7 region of bacterial 16S rRNA genes [15]. The 17 cultivars and growth conditions were also identical to those in [15] (Figure 1). In brief, all greenbugs of each biotype were parthenogenetic descendants from a single female individual and were genetically identical. All plants grew in growth chambers at 25 °C for sorghum or 20 °C for the other species. The populations of greenbug cultures increased and were partially age-synchronized through four 8-day cycles of placement on ‘Newton’ wheat (*Triticum aestivum* L.) with removal of adults after the first two days of each cycle. At the end of the fourth cycle of increase, the different cultivars were then infested with the 7-day to 8-day-old greenbugs and maintained in separate aphid-proof cages for the rest of the 8-day experiment. Because of space constraints, the experiment was conducted first with one biotype and then with the other biotype after disinfecting each of the growth chambers used. The susceptibility to feeding by certain greenbug biotypes other than E was known for 12 of the cultivars, so susceptibility was treated as a fifth experimental variable. All the plants were free of visible disease and did not flower during the experiment.

Each plant was initially infested with an approximately equal quantity of greenbugs. Sampling followed the same schedule for each biotype. The initial, day 0, sample of each biotype was taken from greenbugs on Newton wheat at the end of the fourth cycle of population increase, immediately before the infestation of the different cultivars. Subsequent sampling from the different cultivars happened 2, 4, and 8 days later, which was much less than the 30-day *S. graminum* life expectancy at 19 °C [18]. About 10 to 15 greenbugs were collected per sample, with three samples (biological replicates, hereafter termed replicates) from different plants per biotype per time point per host cultivar. However, the number of individuals per collection ranged from two to twenty due to variation in greenbug survival and reproduction among host plants. Each collection was wrapped in labeled aluminum foil, immediately dropped into liquid nitrogen, and stored at −80 °C. There were 312 samples in total.

### 2.2. Sample Library Preparation and Sequencing

The DNA samples from which ITS sequences were amplified were the same DNA samples from which Crane et al. [15] amplified the V5-V7 region of 16S rRNA genes. The DNA samples had been stored at −80 °C between studies. Crane et al. [15] extracted total DNA from each sample using the DNeasy Blood and Tissue Kit for DNA Isolation (Qiagen, Germantown, MD, USA) according to its instructions. The Internal Transcribed Spacer region 1 (ITS1) was amplified with forward primer ITS1F (CTTGGTCATTTAGAGGAAGTAA) and reverse primer ITS2 (GCTGCGTTCTTCATCGATGC) [19]. This primer pair has been commonly used in fungal metabarcoding research. The MyFi^TM^ DNA polymerase kit (Bioline, Taunton, MA, USA) was used for amplification, producing a 314 bp product. The PCR cycle was as follows: 2 min at 95 °C, followed by 30 cycles of denaturation for 30 s at 95 °C, annealing for 30 s at 55 °C, and extension for 60 s at 72 °C, with a final elongation for 10 min at 72 °C and holding at 4 °C. The amplification was confirmed by electrophoresis on 1.5% agarose gels with SYBR Safe DNA stain (Thermo Fisher Scientific, Waltham, MA, USA).

The 312 amplicon products from the prior ITS1-ITS2 reactions were shipped to RTL Genomics (Lubbock, TX, USA) for quality checking, library preparation, sequencing, and demultiplexing. Illumina overhang adapters and Nextera (Illumina, San Diego, CA, USA) barcode sequences were added to the amplicons using consecutive PCR reactions. The primers for the first reaction were forward (TCGTCGGCAGCGTCAGATGTGTATAAGAGACAG) and reverse (GTCTCGTGGGCTCGGAGATGTGTATAAGAGACAG). The PCR product from the first reaction was the template for the second PCR reaction, which used forward primer AATGATACGGCGACCACCGAGATCTACAC [5-base index]TCGTCGGCAGCGTC and reverse primer CAAGCAGAAGACGGCATACGAGAT [7-base index]GTCTCGTGGGCTCGG. Both PCR reactions used Qiagen HotStar Taq master mix (Qiagen Inc., Valencia, CA, USA), 1 µL of each 5 µM primer, and 1 µL of template in a 25 µL total volume. Both PCR reactions followed initial denaturation at 95 °C for 5 min with 10 cycles of 94 °C for 30 s, 54 °C for 40 s, and 72 °C for 1 min. Both PCR reactions finished with 72 °C for 10 min and were held at 4 °C. The resulting barcoded DNA fragment concentrations were semi-quantitatively scored by fluorescence intensity in eGel gels (Life Technologies, Grand Island, NY, USA) and pooled equimolarly based on gel score. Each pool was size-selected for fragments in the desired length range using SPRIselect beads (BeckmanCoulter, Indianapolis, IN, USA) in a 0.75 ratio of weight to volume. The DNA concentration of size-selected pools was measured using a Qubit 4 Fluorometer (Life Technologies, Carlsbad, CA, USA), and fragment size was confirmed prior to sequencing by an Agilent Fragment Analyzer (Agilent Technologies, Santa Clara, CA, USA). Finally, prepared libraries were loaded at 400 pM and sequenced using an Illumina Novaseq 6000 SP flow cell and PE250 chemistry (Illumina, San Diego, CA, USA). Trimmed, demultiplexed reads were returned in fastq format.

### 2.3. Bioinformatic Analysis

Reads were quality-filtered with deblur [20], which also identified and counted amplified sequence variants (ASVs) using UNITE version 10.0 [8], as released on 19 February 2025 as the reference sequences. Deblur removed all ASVs with fewer than five counts. The ASV file was split by a perl script into 16 parts for parallel identification of taxa by a naive Bayes classifier, unite_ver10_99_all_19.02.2025-Q2-2024.10.qza, imported from https://github.com/colinbrislawn/unite-train/releases (accessed on 16 July 2026). This classifier had been trained on UNITE version 10.0 [8] as released on 19 February 2025. The taxonomic identities were used to purge plant and animal ASVs from the taxon counts and sequences. The remaining fungal ASV counts were collated to genera, and the collated counts were subjected to the Kruskal–Wallis test [21], identification of bimodally distributed relative abundances among samples, network analysis with Weighted Gene Co-expression Network Analysis (WGCNA) [22], identification of differentially populated genera with ancombc [23], calculation of alpha-diversity indices with qiime diversity core-metrics [24,25,26], and PERMANOVA [27] and non-metric multidimensional scaling (NMDS) of Bray–Curtis distances [28,29,30]. The latter calculations were performed with separate sets of counts by genus for each biotype, because plotting relative frequencies indicated a tendency for individual genera to surge in population with biotype K and not with biotype E. All samples were rarefied to 25,750 or 25,760 counts per sample, which retained all samples except one of biotype E.

An R script was written to correlate fungal and bacterial counts by taxon. This script took a table of fungal abundance and a table of bacterial abundance drawn from data used in Crane et al. 2025 [15]. It identified samples present in both tables and taxa that were present in at least 10% of samples. Counts were converted to centered log ratios; a small pseudocount was added to zero values. Covariates were optionally adjusted by residualizing each taxon’s centered log ratio vector on covariates derived from the biotype, host species, host cultivar, and time point. Pearson correlation coefficients were calculated for the Benjamini–Hochberg adjusted (q < 0.01) centered log ratios.

## 3. Results

There were 97,624,609 reads that passed quality control and mapped to 479 fungal or protist entities at the genus level in UNITE (Supplemental Table S1) and 739 entities at the species level (Supplemental Table S2). Other taxa with fewer than five reads had been filtered out by deblur. The ten most abundant entities were “Fungi genus incertae sedis” (21.49% of reads that probably belonged to multiple taxa), *Hannaella* (14.42%), *Fusarium* (5.07%), *Mortierella* (4.58%), *Papiliotrema* (3.90%), *Simplicillium* (2.80%), Debaryomycetaceae genus incertae sedis (2.29%), *Arthrobotrys* (2.28%), Protista genus incertae sedis (2.10%), and Capnodiales genus incertae sedis (2.05%). *Hannaella* consisted mostly of *H. luteola* (9.30%), *H. zeae* (2.63%), and *H. sinensis* (2.43%). *Fusarium* was mainly *F. nygamai* (4.81%) and *F. poae* (0.21%). *Mortierella* species were entirely unidentified. *Papiliotrema* consisted mostly of *P. mangalensis* (3.26%) and *P. flavescens* (0.10%). *Simplicillium* was entirely S. *aogashimaense.* Debaryomycetaceae were not identified to species. *Arthrobotrys* was almost entirely *A. amerosporus*, one of the species that can trap and digest nematodes. The protists were unidentified. The genus Capnodiales incertae sedis was not identified to species, but the order contains sooty molds that thrive on aphid honeydew [31]. Within Capnodiales, major genera *Capnodium*, *Scorias*, and *Phragmocapnias* were not detected explicitly, but *Toxicocladosporium*, *Zasmidium*, and *Cladosporium* respectively constituted 0.11%, 0.0066%, and 0.0014% of reads. Basidiomycete yeasts were common, including not only *Hannaella* and *Papiliotrema* but also *Naganishia* (1.58%), *Rhodotorula* (0.97%), *Cutaneotrichosporon* (0.81%), *Malassezia* (0.39%), *Tausonia* (0.39%), *Rhodosporidiobolus* (0.29%), *Cystofilobasidium* (0.22%), *Vishniacozyma* (0.20%), and *Bullera* (0.08%). The most abundant ascomycete yeasts were Debaryomycetaceae_gen_Incertae_sedis (2.29%), *Trichomonascus* (0.51%), *Candida* (0.40%), *Coniochaeta* (0.26%), and *Pichia* (0.06%). *Saccharomyces* was detected in only eight reads. Other genera of yeasts were rarely detected.

**Figure 2:**
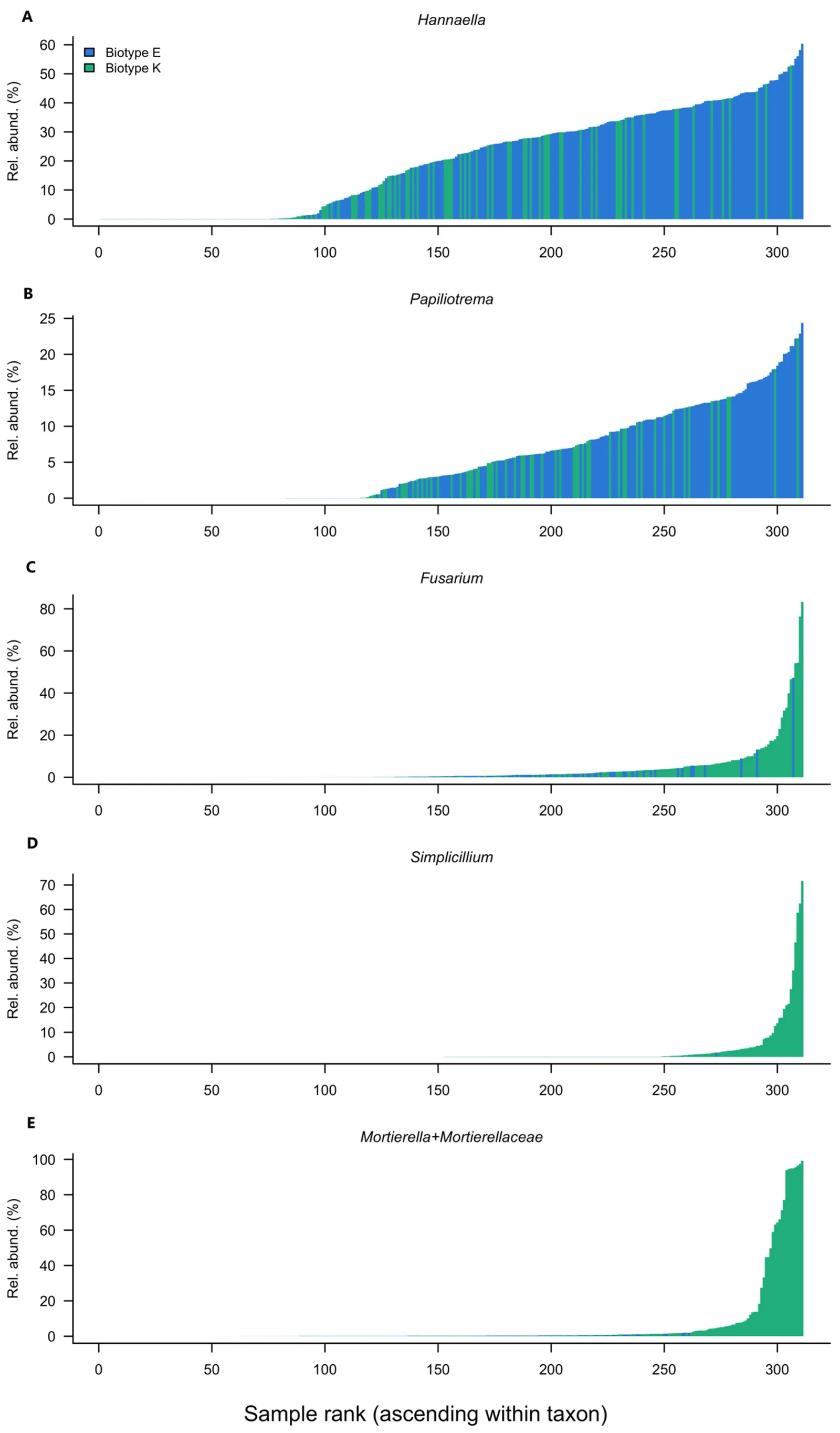
Bar plots of relative abundance of *Hannaella* (**A**), *Papilioitrema* (**B**), *Fusarium* (**C**), *Simplicillium* (**D**), and the sum of *Mortierella* with other Mortierellaceae (**E**), each ordered by its own frequency over 312 samples. (**C**–**E**) depict blooms with high relative frequency in a few samples of biotype K.

**Figure 3:**
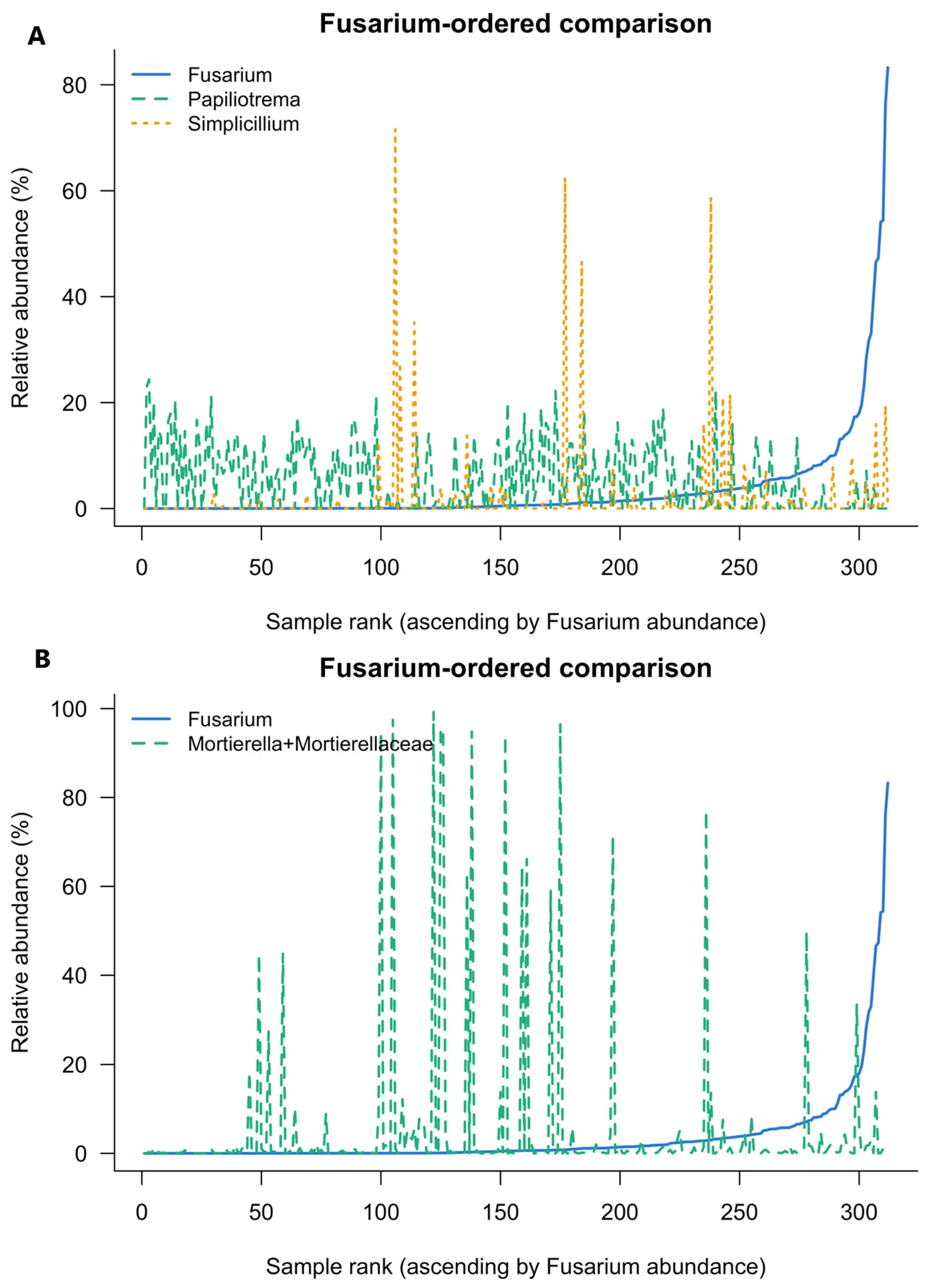
Independently spiking abundance of *Simplicillium* (**A**) and *Mortierella* with Mortierellaceae (**B**) versus relative abundance of *Fusarium*. Relative abundance of *Papiliotrema* is relatively constant but negatively correlated with *Fusarium* (Spearman’s rho = −0.343, *p* = 4.9 × 10^−10^) because the highest fractional abundances of *Fusarium* arithmetically displace everything else.

Ranking samples by relative frequency of *Hannaella* revealed a continuous gradation of frequency and no visually obvious association of abundant *Hannaella* with biotype (Figure 2A). *Papiliotrema* (Figure 2B) also graded continuously upward but was more abundant in biotype E. In contrast, other prominent genera were abundant in small subsets of samples and mostly limited to biotype K (Table 1; Figure 2C–E). However, peak relative frequencies of the prominent genera were not visibly associated with one another (Figure 3A,B), even though rank correlations among them were often significant. For Figure 3, Spearman’s rho was −0.343 for *Papiliotrema* with *Fusarium* (*p* = 4.9 × 10^−10^), +0.341 for *Simplicillium* with *Fusarium* (*p* = 6.1 × 10^−10^), +0.247 for Mortierellaceae with *Fusarium* (*p* = 1.0 × 10^−5^), and +0.110 for *Mortierella* with *Fusarium* (*p* = 0.052). Weighted gene co-expression network analysis (WGCNA) [22] identified five modules of correlated genera in biotype E and six modules in biotype K. Their rosters appear in Table 2. “Blue” modules with *Wallemia* and *Fusarium* intersected between biotypes E and K, although the module included more genera in biotype K. “Brown” modules with *Hannaella*, *Papiliotrema*, and *Candida* also intersected between biotypes E and K and included more genera in biotype K.

After rarefaction to 25,750 counts, PERMANOVA was run separately for biotypes E and K on Bray–Curtis distances between genera grouped by module. Module membership accounted for 17.7% of variance in biotype E (*p* = 0.001) and 10.4% of variance in biotype K (*p* = 0.001). PERMDISP revealed lower dispersion in the *Hannaella* module of biotype E (0.50 versus ca. 0.65 in other modules).

The Raup-Crick test in the R package vegan [32] was run for each biotype with genera as objects to test if genera within modules were more likely to co-occur in individual samples than would be expected by chance. The result was significant (R^2^ = 0.262 for E and 0.534 for K, *p* = 0.001 for both biotypes), but dispersion was greater in biotype E, ranging from 0.097 to 0.650 among modules, versus 0.170 to 0.385 among modules in biotype K.

The fungal microbiota associated with biotypes E and K on ‘Newton’ wheat at day zero were similar but not identical in composition. *Hannaella* was the most abundant genus in all six replicates and ranged from 26.8 to 47.9% of total counts. *Papiliotrema* ranked second in four replicates and third in the other two; it had 11.3 to 14.1% of total counts. Unidentified Microbotryomycetes ranked third, fourth, or fifth among all six replicates. However, Capnodiales *genus incertae sedis* ranked second in one replicate and no higher than fifth elsewhere, and *Mariannaea* ranked second in one replicate but was not otherwise in the top ten. The sum of the 470 genera that ranked below tenth ranged from 3.4 to 11.6% of total counts.

**Figure 4:**
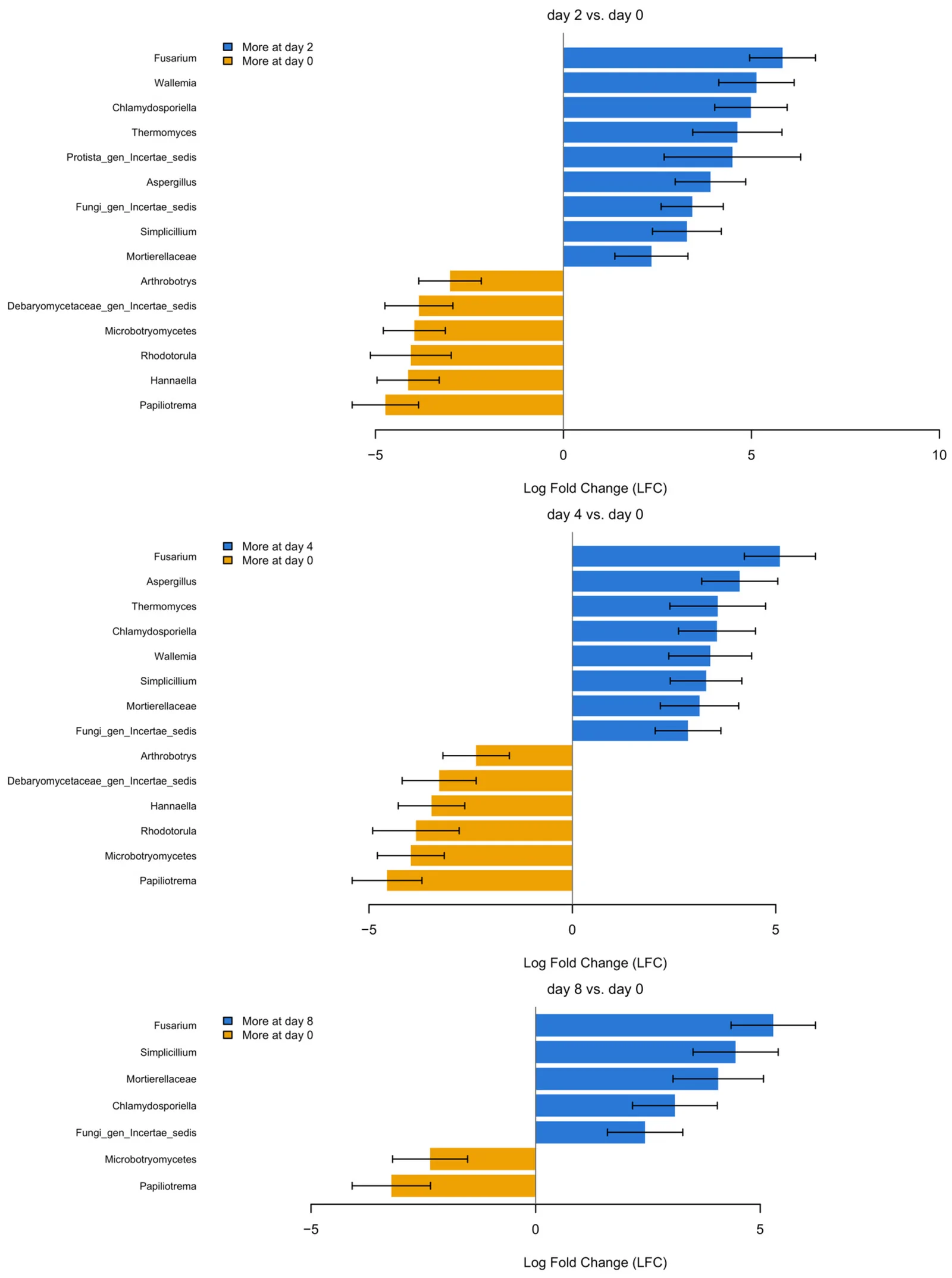
Bar plots of log_2_ fold-change at three time points for the most responding genera in biotype K as identified by ancombc [23]. The minimum eligible absolute value of log2 fold-change was 2.

The frequency of samples with dominant *Mortierella* and Mortierellaceae increased with time. The percent of samples with greater than 5% *Mortierella* counts was 2.0% at day 2, 5.0% at day 4, and 15.7% at day 8, with *p* = 0.0013. The corresponding percentages for otherwise unidentified Mortierellaceae were 1.0, 5.0, and 12.7, with *p* = 0.0045, and 28.4% of day-8 samples had at least 5% Mortierellaceous counts. Other genera did not exhibit this proliferation over time with the Kruskal–Wallis test applied to sample counts with greater than 5% relative abundance, but they did appear significant when ancombc [23] was applied to read counts directly. With biotype K, filamentous fungal genera *Fusarium*, *Simplicillium*, *Chlamydosporiella*, and unidentified Mortierellaceae generally increased over the time course at the expense of yeasts *Hannaella* and *Papiliotrema* (Figure 4). In contrast, no genus significantly increased or decreased over the time course with biotype E. More genera were significantly correlated with time when rank-correlations were calculated directly from relative abundance in the count files without rarefaction (Table 3). With biotype K, the negative correlations outnumbered the positive correlations by 36 to four. No genus significantly increased or decreased with rank correlations for biotype E.

**Figure 5:**
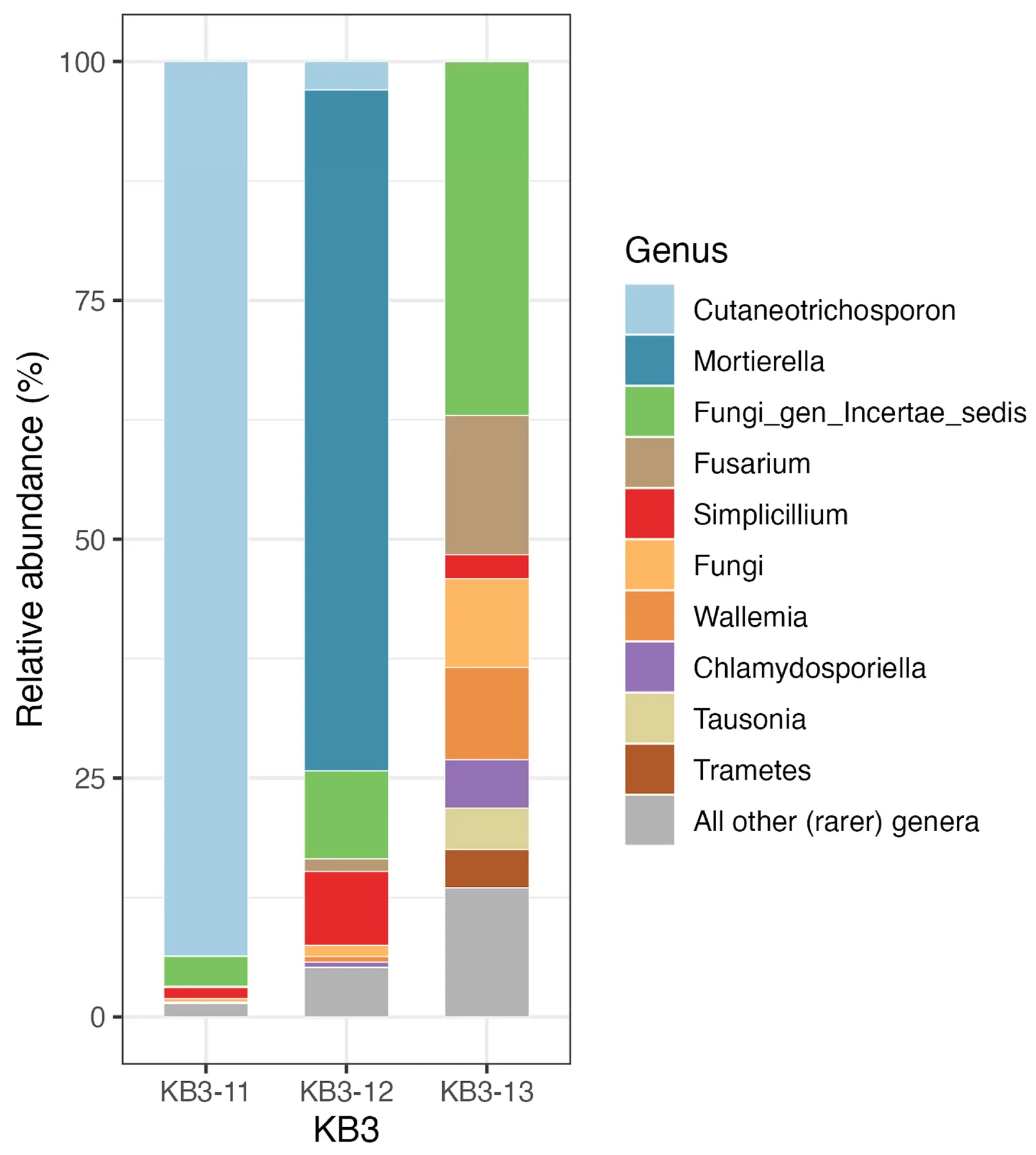
Widely differing dominant genera among sister replicates of biotype K collected from barley cultivar Post 90 on day 2. The bar labels are codes for biotype (K), species (barley, B), cultivar (Post 90, coded as 3), time (day 2, coded as 1), and replicate (the last digit in the code).

Sister replicates frequently differed greatly in taxonomic composition (Figure 5). For example, there were three replicates of biotype K on barley at day 2, totaling 342,657, 334,000, and 296,263 counts, respectively. The first replicate was dominated by *Cutaneotrichosporon* (93.6% of total counts), “Fungi genus incertae sedis” (3.1%), and *Simplicillium* (1.2%). The second replicate was dominated by *Mortierella* (71.3%), “Fungi genus incertae sedis” (9.2%), and *Simplicillium* (7.7%). The third replicate was dominated by “Fungi genus incertae sedis” (37.0%), *Fusarium* (14.6%), and *Wallemia* (9.7%). The 10 most abundant genera accounted for 99.0, 96.1, and 90.1% of all counts in these respective replicates. This is another example of blooms by single taxa in biotype K.

**Figure 6:**
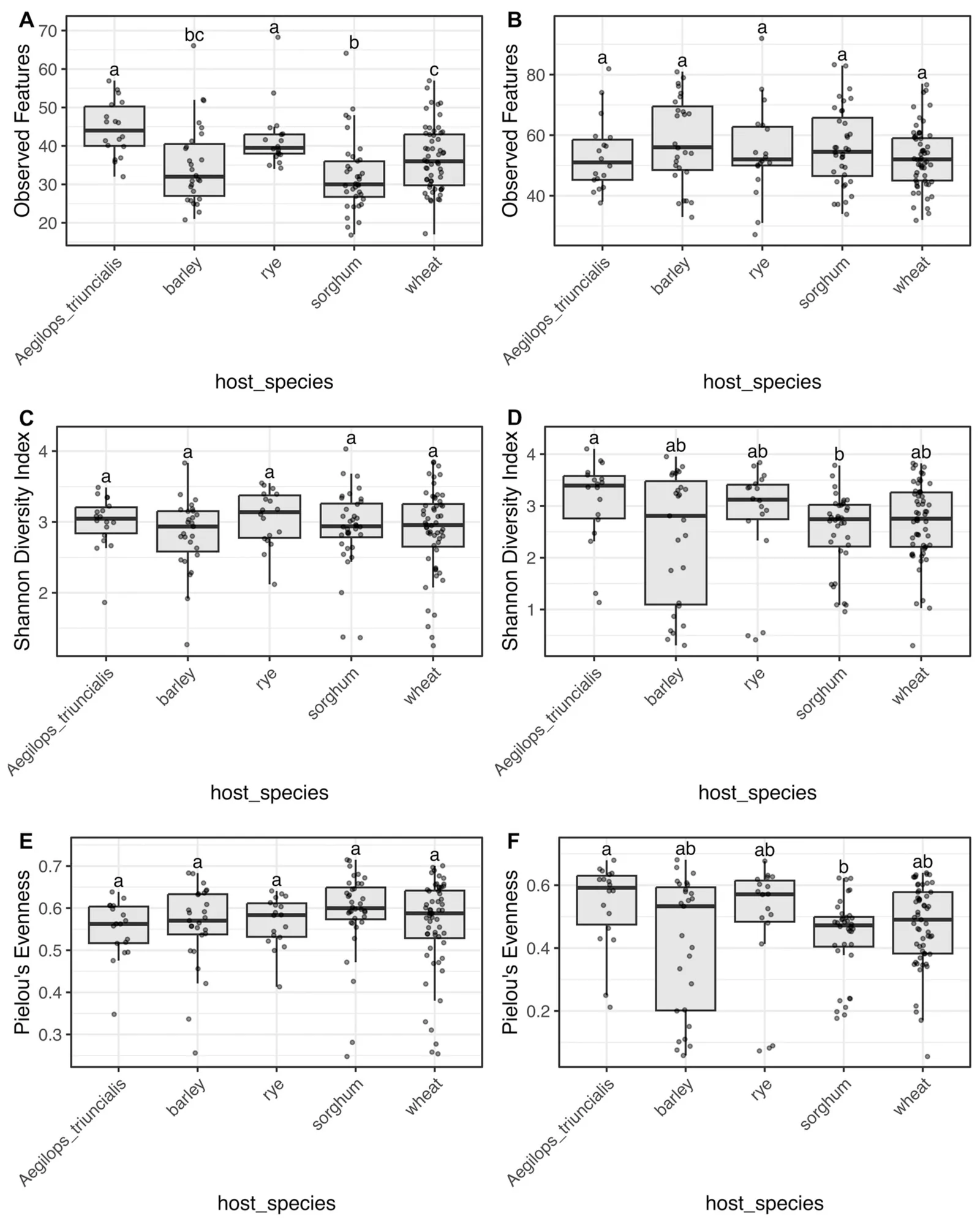
Comparison of alpha diversity of fungi among host plant species infested with greenbug biotype E (**A**,**C**,**E**) or biotype K (**B**,**D**,**F**). The heavy, horizontal bar within each box is the median. Boxes extend from the 25th to 75th percentile of values. Whiskers extend to the farthest data point within 1.5 times the interval between the 25th and 75th percentiles. Jittered dots are the values of the index in each plot. Letters above each box indicate Benjamini–Hochberg adjusted significance at 5%; multiple letters indicate that the species does not differ significantly from the two extreme species.

**Figure 7:**
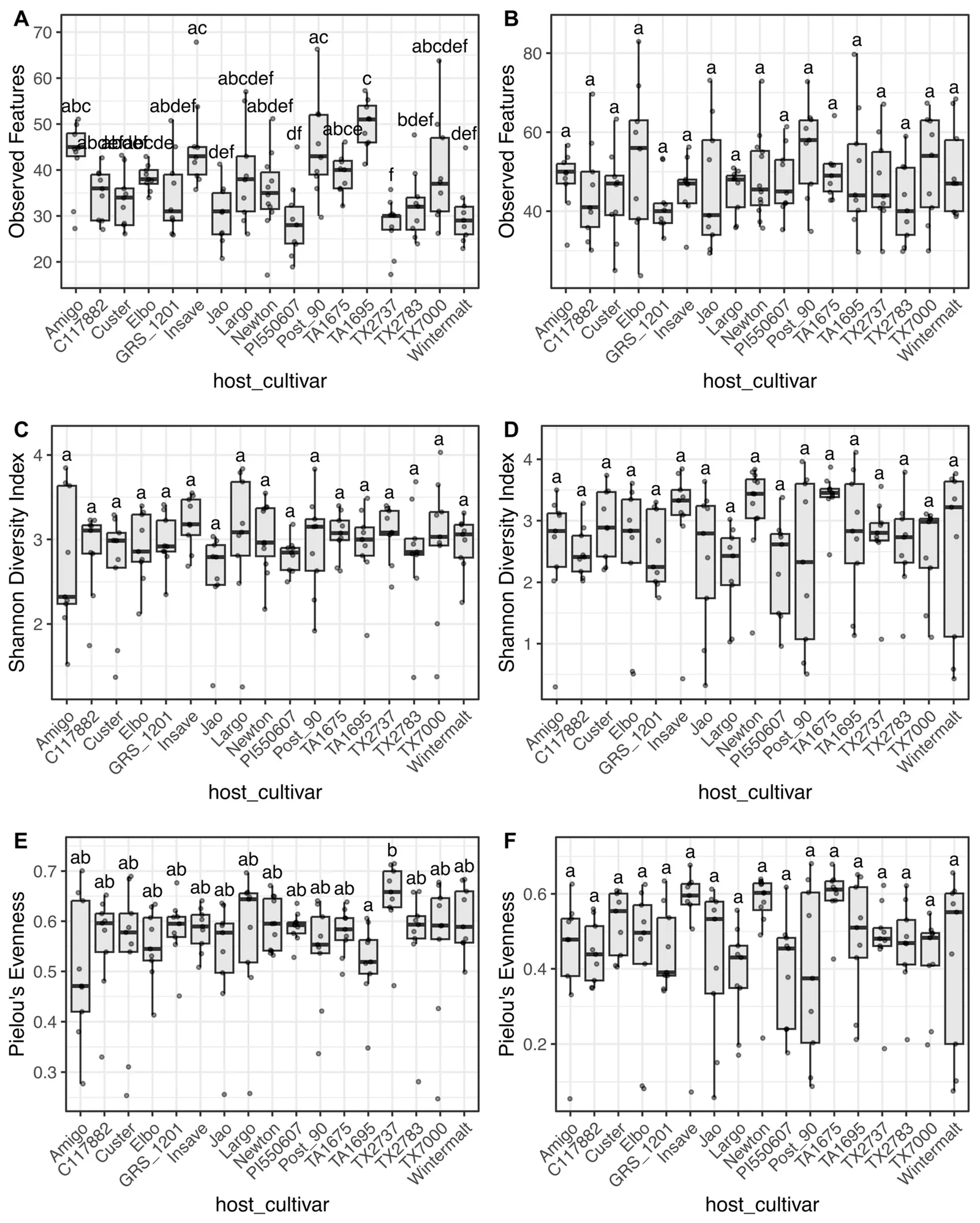
Comparison of alpha diversity of fungi among host plant cultivars infested with greenbug biotype E (**A**,**C**,**E**) or biotype K (**B**,**D**,**F**). The heavy, horizontal bar within each box is the median. Boxes extend from the 25th to 75th percentile of values. Whiskers extend to the farthest data point within 1.5 times the interval between the 25th and 75th percentiles. Jittered dots are the values of the index in each plot. Letters above each box indicate Benjamini–Hochberg adjusted significance at 5%; multiple letters indicate that the cultivar does not differ significantly from the two extreme cultivars.

**Figure 8:**
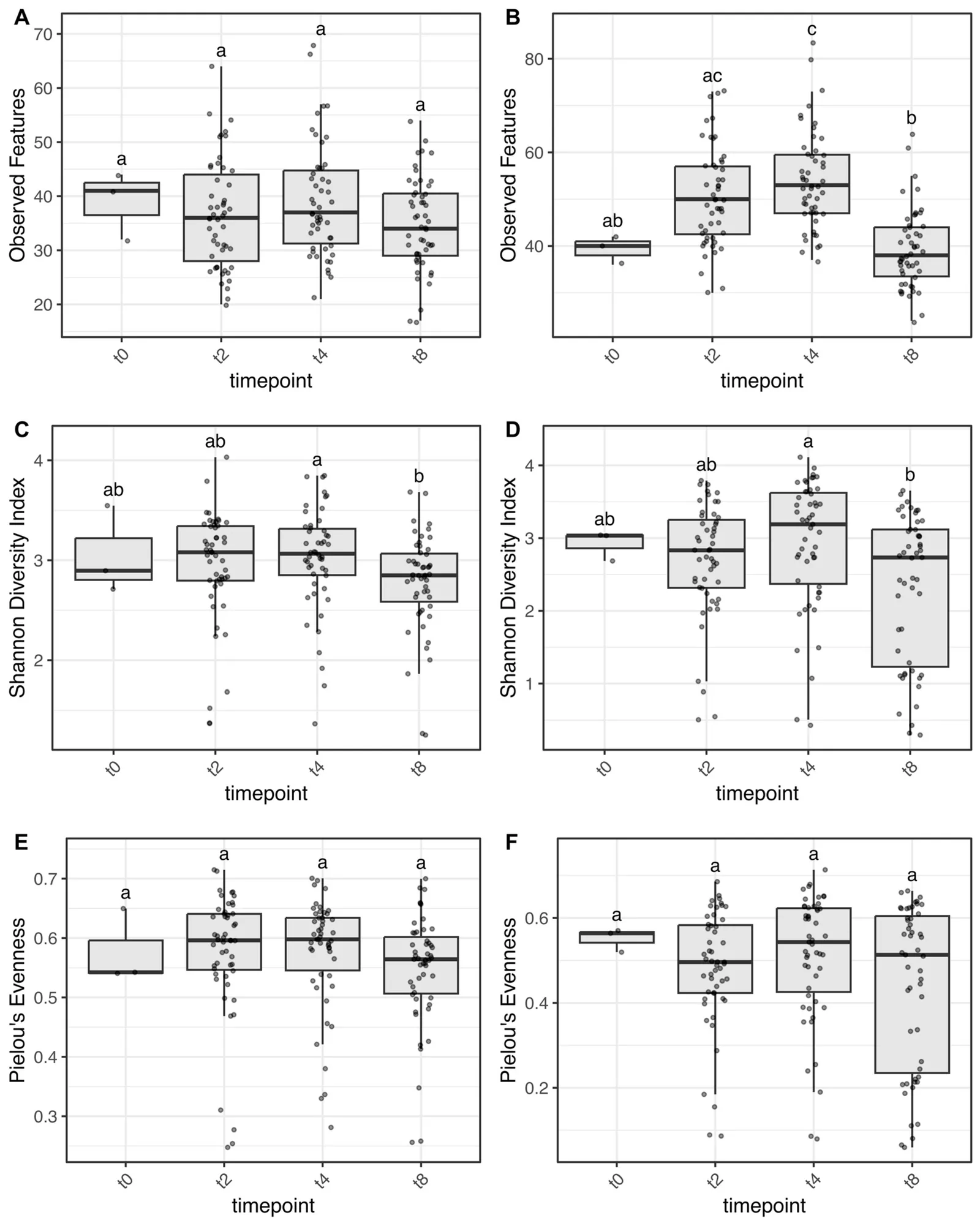
Comparison of alpha diversity of fungi among sampling dates with infestation by greenbug biotype E (**A**,**C**,**E**) or biotype K (**B**,**D**,**F**). The heavy, horizontal bar within each box is the median. Boxes extend from the 25th to 75th percentile of values. Whiskers extend to the farthest data point within 1.5 times the interval between the 25th and 75th percentiles. Jittered dots are the values of the index in each plot. Letters above each box indicate Benjamini–Hochberg adjusted significance at 5%; multiple letters indicate that the sampling date does not differ significantly from the two extreme sampling dates.

Alpha diversity of the fungal communities was measured separately for biotypes E and K in three ways: observed features, Shannon entropy, and Pielou’s evenness. Samples were rarefied to 25,760 reads for both biotypes. This level of rarefaction did not recover all detected genera, but it fully saturated diversity indices. Among host plant species, there were more observed fungal genera with biotype K than with biotype E, but there was less significant variation among the host plant species with biotype K (Figure 6A,B). Also, the ranking of fungal genera among the host plant species differed between the two biotypes. Except in *Aegilops*, Shannon entropy was slightly lower with biotype K, but the only significant difference among the host species was between *Aegilops* and sorghum with biotype K (Figure 6C,D). Evenness was also lower with biotype K, except on *Aegilops*, and again the only significant difference was between *Aegilops* and sorghum with biotype K (Figure 6E,F), with sorghum being lower for both indices. Among host cultivars, observed fungal genera were fewer with biotype E than with biotype K, but biotype E had a complex pattern of significant differences among the cultivars, with extremes of 51 genera in *Aegilops* Ta1695 and 27 genera in PI550607 (Figure 7A). In contrast, *Aegilops* Ta1695 had the fewest observed fungal genera with biotype K, and despite visible variation, the cultivars did not differ significantly from one another with biotype K (Figure 7B). Shannon diversity did not vary significantly among host cultivars with either biotype (Figure 7C,D), although the extreme values were farther apart with biotype K. Evenness among cultivars was lower with biotype K than E, but only the difference between *Aegilops* Ta1695 and sorghum TX2737 with biotype E was significant (Figure 7E,F). With collection dates, there were again fewer observed taxa with biotype E than with biotype K, and biotype K had a significant increase in observed taxa through day 4 followed by a decrease at day 8 as blooming taxa took up a greater share of counts (Figure 8A,B). Shannon entropy was greatest at day 4 for both biotypes before decreasing at day 8, and the values were similar between biotypes (Figure 8C,D). Evenness was lower with biotype K and fell from day 4 to day 8 in both biotypes, but the difference was not significant (Figure 8E,F).

**Figure 9:**
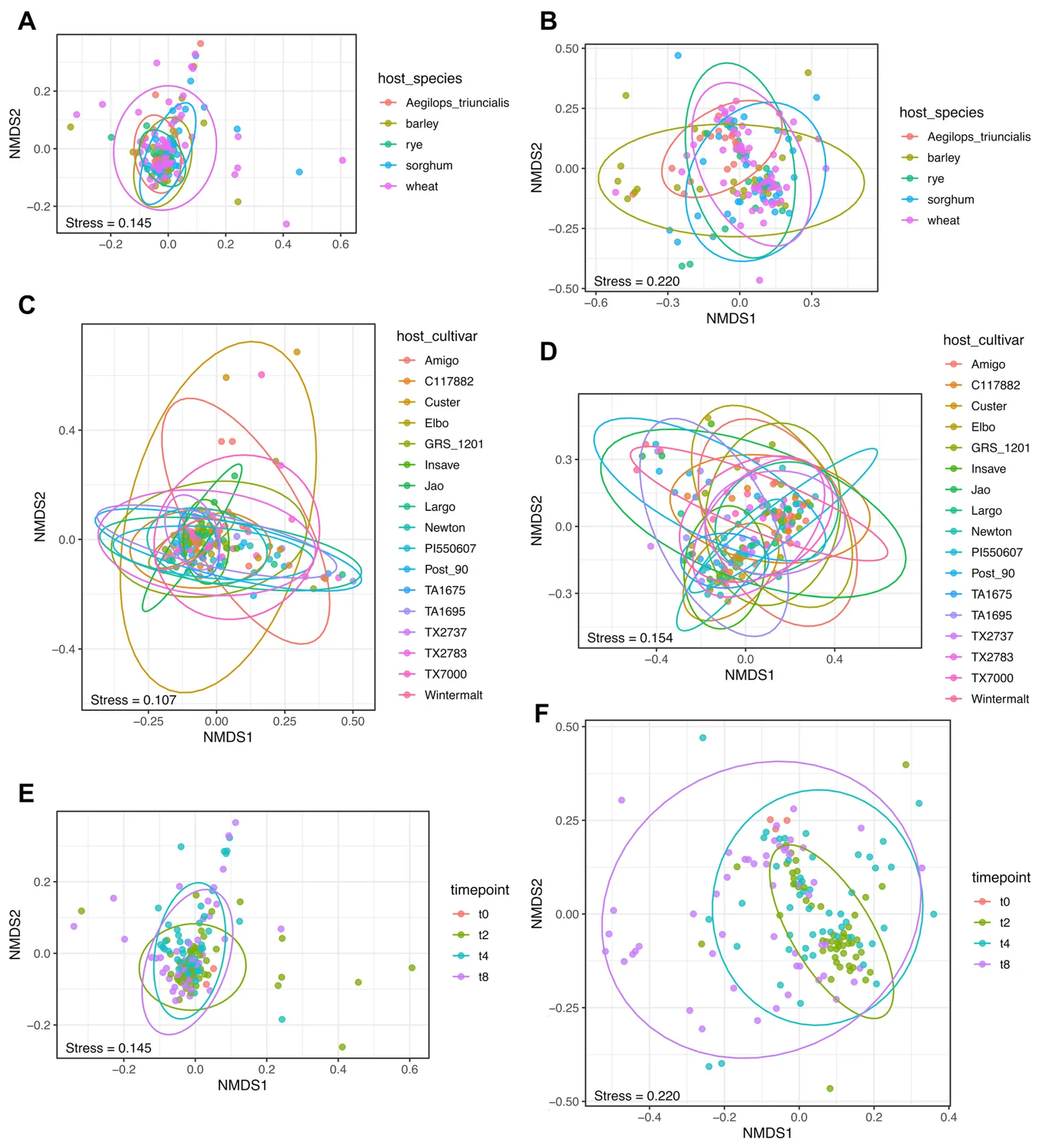
Non-metric multidimensional scaling of Bray–Curtis distances by host species, host cultivar, and sampling date. (**A**) Host species with biotype E; (**B**) Host species with biotype K; (**C**) Host cultivar with biotype E; (**D**) Host cultivar with biotype K; (**E**) Time point with biotype E; (**F**) Time point with biotype K.

Beta-diversity (diversity among samples) was investigated with Bray–Curtis distances, which were subjected to PERMANOVA with adonis2 (Table 4) [33] and non-metric multidimensional scaling (Figure 9) [28]. For Table 4, PERMANOVA was conducted separately with models containing host species and host cultivar, because cultivars could vary independently. Factors were biotype, time point, host species, host cultivar, and host susceptibility to greenbug feeding, ranked from most to least significant. The first four factors and most of their mutual interactions were highly significant with *p* ≤ 0.0002. Susceptibility was not significant. The ranking was robust to varied order of factors in the model. Biotype was overwhelmingly the most important single factor, with R^2^ seven times greater than for time point. The interaction of biotype and time point was more significant than time point itself, which reflected the tendency of individual taxa to bloom over time in biotype K. The R^2^ value was also consistently greater for time point than for host species or cultivar when the biotypes were analyzed separately. The R^2^ values for biotype K were greater than for biotype E in the separate analyses, indicating a greater responsiveness of biotype K’s fungal microbiota to the host plant.

Non-metric multidimensional scaling was performed separately for each biotype with regard to host species, cultivar, and time point. The displays in Figure 9 include points for the individual samples and ellipses that are intended to enclose 95% of the distribution of points. For species with biotype E, wheat had the greatest dispersion of points and therefore the most varied communities, while *Aegilops triuncialis* had the least (Figure 9A). With biotype K, rye had the greatest dispersion and sorghum the least, and barley responded differently from the other species, since its ellipse was orthogonal to the others (Figure 9B). For cultivars with biotype E, Custer had the greatest dispersion and most ellipses overlapped, which indicated relative stability of the fungal microbiota across cultivars (Figure 9C). With biotype K, the overlap pattern was complex, but there appeared to be three groups of cultivars with roughly aligned major axes (Figure 9D). For time points with biotype E, the displayed ellipses tightly overlapped, although seemingly more than 5% of the points were outside of them (Figure 9E). The tight overlap indicated a stable fungal microbiota over time. With biotype K, the expanding ellipses indicated a vigorously diversifying set of communities as individual taxa bloomed over time (Figure 9F).

**Figure 10:**
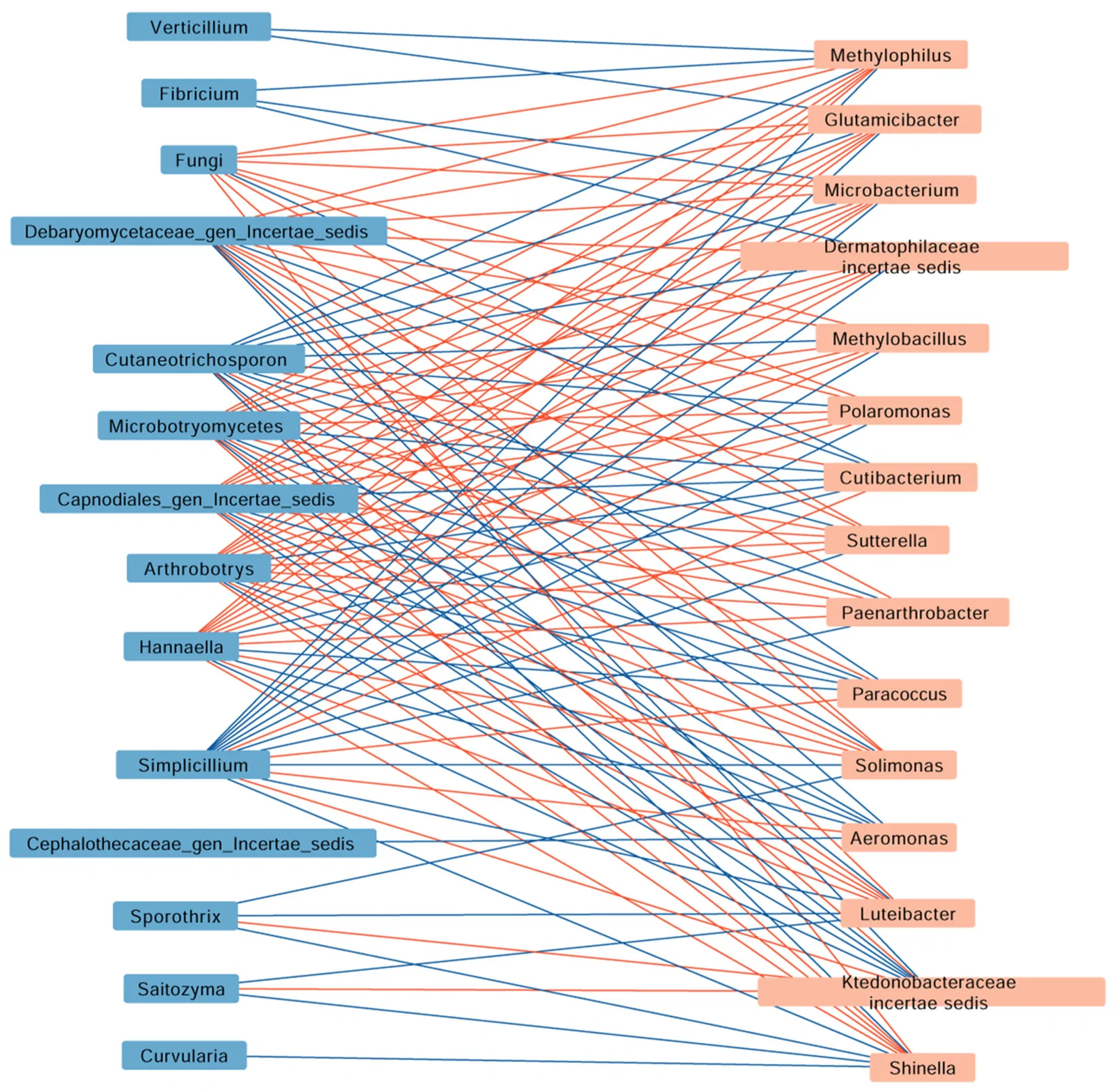
Positive (blue edges) and negative (orange edges) correlations between top 15 fungal (**Left**) and top 15 bacterial (**Right**) genera. Top was determined by the number of significant (q < 0.01) correlations to other taxa.

The bacterial frequencies of Crane et al. [15] and the fungal frequencies from the current study were based on separate amplifications from the same DNA samples, which allowed frequency correlations between fungal and bacterial genera. For the top 15 fungal and top 15 bacterial genera as ranked by count of significant correlations, Figure 10 presents 126 correlations that were significant at q < 0.01. Negative correlations outnumbered positive correlations, 71 to 55. Taxa from the *Hannaella* module of Table 2 were mostly negatively correlated with bacteria. *Simplicillium* and *Cutaneotrichosporon* were mostly positively correlated with these same bacteria. The significant bacterial correlations of *Verticillium*, *Fibricium*, unidentified Cephalothecaceae, and *Curvularia* were exclusively positive. The bacterial correlations of *Sporothrix* and *Saitozyma* were positive, except for unidentified Ktedonobacteraceae. None of the depicted bacterial genera were exclusively positively or negatively correlated with the depicted fungal genera, but *Aeromonas* and *Cutibacterium* were mostly positive, and *Polaromonas*, *Methylobacillus*, and *Sutterella* were mostly negative.

## 4. Discussion

An exploratory survey was conducted with ITS sequences to investigate the effects of aphid biotype, host plant species, host plant cultivar, host plant resistance to greenbug feeding, and elapsed time over an eight-day time course on the composition and diversity of the fungal microbiota associated with greenbug aphids. The analysis was based solely on frequency data, which limited what could be concluded about the mechanisms responsible for the observed effects.

Biotype affected diversity more than time point, which elicited a slightly stronger response than either host species or host cultivar, but all four of these factors were significant, as were interactions of biotype with these three factors. In contrast, host plant resistance to greenbug feeding did not elicit a significant response, possibly because cultivars were classified as resistant based on published responses to other greenbug biotypes, but biotype E has overcome this resistance [1]. Basidiomycete yeasts were abundant, but other taxa often bloomed to dominance, especially in biotype K. Analysis of frequency correlations with weighted gene co-expression network analysis (WGCNA) [22] identified six modules of 10 to 28 correlated genera in biotype K and five modules in biotype E. Members in each module were more likely to co-occur in the same sample than would be expected by chance with the Raup-Crick test, which is consistent with sampling from distinct communities, especially in biotype K.

Aphid pathogens were rare in the fungal microbiota. Only three facultative aphid pathogens, *Fusarium solani*, *Fusarium oxysporum*, and *Trichoderma longibrachiatum*, were detected, with a total of 1276 counts over a total of eight of the 312 samples, and all three of these species can live saprophytically. The most frequent *Fusarium*, *F. nygamai*, has been shown to cause foot rot of rice [34], but it is not a known pathogen of either aphids or the five host plant species in our study.

The fungi encountered here differed from the 253 genera reported by Crane et al. (2025) [14] for mRNA-derived reads. Only 67 of these genera were encountered in our current study, and *Gilbertella* was the most abundant there but absent here. Three of the genera were plausibly in close contact with greenbugs: *Malassezia*, which is often on animal skin; *Mucor*, which has been found in digestive tracts; and *Mycotypha*, which includes a gut symbiont of silverfish [35]. Notably missing in the mRNA study were *Mortierella*, *Simplicillium*, *Arthrobotrys*, and among yeasts, *Papiliotrema*, *Naganishia*, *Tausonia*, *Rhodosporidiobolus*, *Cystofilobasidium*, *Vishniacozyma*, and *Bullera*. Apart from actual differences in abundance, there are three obvious reasons for these missing genera. First, there were only 571 fungal genomes in the dataset of Crane et al. [14] versus 266,589 ITS sequences in UNITE, and coverage of fungal orders with sequenced genomes was likely uneven. Second, bacterial reads greatly outnumbered fungal reads in the mRNA study, so rarer fungi did not reach the threshold of 100 total counts to be considered in that study. Third, fungal nomenclature has been in flux since the application of DNA sequence data to taxon definitions, so there could be additional taxa in common between the two studies once the synonymy has been reconciled. Still, it is concerning that *Gilbertella* was the most abundant genus in the mRNA study and completely absent in the current study. *Gilbertella* was separated by Hesseltine [36] from *Choanephora*, which is also absent in the current study. *Gilbertella persicaria* has been mated with *Rhizopus stolonifer* [37], and it is possible that the taxon that matched *G*. *persicaria* in the RNA study was mapped to *Rhizopus arrhizus* in the current study. However, *R. arrhizus* was rare in the current study, and *G. persicaria* was common in the mRNA study. Abundance suggests that *Mortierella* might be a better match in the current study, but no *Mortierella* have sequenced genomes yet. The abundance of “Fungi class incertae sedis” and unidentified “Fungi” in Figure 2 suggests that the *Gilbertella* in the mRNA study might map to either of them in the current study, if there is a high enough fraction of rRNA reads in the former to hit UNITE.

Wolfgang et al. [38] found variation in microbial taxon presence between individual oak aphids and argued on this basis that aphids should not be pooled for microbiota analysis. However, Wolfgang et al.’s amplification with fungal primers frequently failed with single aphids. The mass of individual aphids varied among cultivars in our study, possibly reflecting the effect of known resistance genes to aphid feeding on growth rate, so a naïve count would not have equalized sequencing depth. Our sampling took what it could get.

As in Crane et al. 2023 and 2025 [14,39], greenbugs were sampled by freezing an infested leaf segment in liquid nitrogen and then brushing the aphids off. This avoided injury to the greenbugs and did not compromise that RNA-seq experiment [14], since all of their reads were mapped to greenbug, wheat, and a battery of ca. 63,000 microbial genomes before further analysis of the reads that most closely matched the greenbug genome. However, this sampling method contaminated the greenbug samples with nucleic acids from previously excreted honeydew and uninfested parts of the leaf, because the frozen leaf tissue was very brittle. The mRNA-based reads in [14] mapped 97.6% to greenbug, 1.2% to wheat, and 1.1% to microbial genomes. In the current study with ITS amplicons, there was no way to filter out contaminating reads from the greenbugs’ immediate environment, and ITS sequences could have been amplified to high levels from fungi in the immediate environment if there were few fungi within or bound to the greenbugs.

Our most fundamental hypothesis was that greenbugs have a fungal microbiota. A diverse fungal microbiota was detected, but in the absence of microdissection or microscopy of the gut and cuticle, it is not possible to confirm that this fungal microbiota was in the greenbugs. Three factors strongly militate against the existence of a gut fungal microbiota in greenbugs. First, the internal diameter of the aphid stylet (0.7 to 2 µm; [40,41] is too small to pass typical yeast cells (3 to 6.5 µm in diameter; [42,43]. Second, aphids lack the musculature to aspirate external matter through the anus into the hindgut [44]. Third, while aphids do not excrete continuously, they do excrete frequently and can output a third of their body mass in honeydew per hour [45], aided by a 10-bar gradient in hydrostatic pressure from sieve tubes to anus while feeding [46]. The implication is that aphids cannot swallow fungal cells and would rapidly excrete any microbes that do not adhere to the gut lining, which is lined with ca. 1.6 µm microvilli that appear to be too short to trap 3 × 6 µm yeast cells [47]. Published micrographs from the empty gut of other aphids support the idea that the gut does not contain fungi (Figure 2 of [47]; Figure 13 of [48]). There are conflicting reports that fungi could [9] or could not [49] be isolated from the aphid gut, although Schanderl et al. [49] also reported that budding yeast cells appeared after 12 h of culture of *Myzus persicae* and *Aphis pomi* guts in hanging drops. The unsolved issue is contamination from the outside; specifically, whether the surface sterilization method of [9] (96% ethanol for 3–4 min, 4% bleach for 1 min, and rinsing in sterile water) was sufficient to remove fungi from the aphid cuticle, and how effective their subsequent sterile technique was.

Statistical analysis upheld the second hypothesis, that the fungal microbiota would respond to the experimentally varied aphid biotype, host plant species and cultivar, and elapsed time. Both composition and diversity indices responded, reflecting frequency shifts for individual taxa and modules of similarly responding taxa. Greenbug biotype had much more influence than the other experimental variables.

The observed taxon frequencies did not support the third hypothesis, that RNA-seq and ITS sequencing would identify the same taxa. The caveat is that each method was applied to a different pair of greenbug biotypes in the same growth chambers, and sequences were mapped to databases that differed greatly in depth of fungal taxonomic coverage. Also, fungal genomes vary 100-fold in the copy number of 18S rRNA sequences [50], and basidiomycetes generally have more copies than ascomycetes do, which would have led to an overcount of basidiomycetes. Nevertheless, the failure to recover *Gilbertella*, the leading fungal genus in [14], in the current study is disconcerting.

Many of the basidiomycete genera found in the current study were also abundant on leaf surfaces of maize that were not infested with any insects or mites [51], so there is a strong relationship between the greenbug-associated fungal microbiota and the uninfested phylloplane fungal microbiota. Nevertheless, greenbug biotype, not host plant, most strongly influenced differences in the composition of the fungal microbiota between samples. Without an aphid-free control for each cultivar, it is not known how much the phylloplane fungal communities differed from one another before infestation. Greenbug arrival at a new plant introduces honeydew to the phylloplane. The honeydew is an initially eutrophic environment that slowly dries to a sticky syrup while microbes deplete its nutrients. Thus, it is plausible that fresh and old honeydew support different end-point communities, that there is a smooth transition to the old-honeydew community as the honeydew ages, and that the community can be rejuvenated when new honeydew is excreted over old honeydew. The phylloplane is an initial source of inoculum for honeydew, but aphids come into contact with honeydew as they crawl around on the leaf and therefore can carry microbes from old honeydew to fresh honeydew. The old honeydew is likely enriched in organisms that thrive in honeydew, when compared to random taxa on the virgin phylloplane, and therefore aphid movement can in principle maintain a community of honeydew-adapted taxa across aphid generations and from one host plant to the next. An alternative hypothesis is that the different aphid biotypes produced different honeydew compositions from the same diet and that these differences favored different taxa in the honeydew. In either case, the day-0 fungal microbiota did not completely specify the fungal microbiota in any subsequent sample, and the feedback interactions that allowed large deviations from day-0 in some samples remain unknown.

Since honeydew is excreted as discrete droplets, the honeydewed phylloplane is covered with a patchwork of droplets of different ages, each potentially with its own taxonomic frequencies. This community differentiation would lead to negative correlations in frequency between the dominant taxa in different droplets, and these negative correlations would strengthen over time. Actively antagonistic interactions, whether competitive or allelopathic, could also result in negative correlations between taxa. In this regard, it is worth noting that at least five genera in the *Hannaella* module, including *Hannaella*, *Papiliotrema*, *Coniochaeta*, *Hansfordia*, and *Arthrobotrys*, produce antifungal or antibacterial compounds, and the *Hannaella* module of biotype K had the highest fraction of negative correlations in frequency with other taxa. *Hannaella sinensis* has been proposed as a biocontrol agent for *Penicillium* spoilage of apples [52]. *Papiliotrema laurentii* can inhibit spoilage by various fungi on fruits [53], and *Papiliotrema flavescens* emits acetoin, naphthalene, and indole [54]. *Coniochaeta hoffmannii* produces coniotins A, B, C, and D, which inhibit the growth of *Candida* and *Aspergillus* [55]. *Coniochaeta velutina* produces anthraquinone glycosides [56]. *Hansfordia* produces 13-deoxyphomenone [57]. *Arthrobotrys* produces antibacterial sesquiterpene polyketides [58] and directly parasitizes *Rhizoctonia solani*, a plant pathogen [59]. In the *Wallemia* module of biotype K, *Fusarium nygamai* is a member of the *F. fujikuroi* complex [60] and thus a potential fumonisin producer; fumonisins can inhibit the growth of other fungi [61]. Dead aphids probably did not contribute a distinct community to the fungal microbiome, because the different cultivars were infested with seven- to eight-day-old greenbugs that were early in their 30-day life expectancy [18].

Our experiment would have benefited greatly from a different sampling method and two types of controls. Honeydew contamination would have been less if aphids had been brushed off the leaf into a microcentrifuge tube before flash-freezing, because leaf fragments would not have been sampled. However, the standard practice of surface-sterilizing the aphids with ethanol and hypochlorite bleach likely would not have eliminated contaminating DNA from the aphid cuticle, even if external organisms were killed. McGreig et al. [62] found that a two-second ethanol immersion followed by one minute in bleach and rinsing in deionized water did not fully remove experimentally applied *Lilium* pollen from spittlebugs as detected by PCR with plant-specific primers. Waxy *Lilium* pollen is less sticky than dried-on honeydew. One obvious control for phylloplane or honeydew contamination would have been to save the leaf pieces after brushing off the aphids and amplifying ITS sequences from them. This would have been logistically difficult, given their brittleness, but would have allowed any aphid-specific taxa to be recognized. A second control to document ecological succession in honeydew would have been to allow aphids to feed and excrete on leaves for a short time, then remove the aphids and sample the honeydewed leaf tissue over the time course for ITS amplification. Microscopic examination of freshly excreted honeydew could have provided direct evidence for or against the presence of a gut microbiota. Honeydew could have been sucked into a glass capillary during excretion, then expelled onto a hemocytometer slide and examined with differential interference-contrast (Nomarski) optics to census bacterial or fungal cells in a known volume. Scanning electron microscopy (SEM) of the gut lining could detect if a bacterial biofilm was present, while wet SEM of the cuticle could document contaminants there. All of these recommendations apply to future research; it is not possible to rectify their absence retrospectively here. Finally, if all fungi truly were derived from the phylloplane and honeydew, a perfect removal of cuticular contaminants would have precluded amplification of any ITS sequences from the aphids.

## 5. Conclusions

In conclusion, visibly healthy greenbugs supported in themselves or their immediate environment a diverse fungal community with abundant basidiomycete yeasts. Greenbug biotype, elapsed time, and host plant genotype influenced fungal species composition in decreasing order. Host plant resistance to greenbug feeding had little effect. Replicate samples could differ drastically in dominant taxon, perhaps because of unintended subsampling from spatially distinct communities. The different community compositions between biotypes possibly reflected different compositions of honeydew derived from the same diet, or carryover of inoculum in honeydew that adhered to the greenbug cuticle. Fungal diversity decreased with elapsed time as successful taxa apparently outcompeted the rest. Five modules of correlated genera were identified in biotype E, and there were six such modules in biotype K. These modules consist of 10 to 28 genera and might represent distinct fungal communities. A *Hannaella* module was negatively correlated with most other fungi and bacteria, and at least five of its genera are known to produce antifungal or antibacterial substances. Taxonomic abundances estimated from ITS read counts differed from abundances assessed by RNA-seq against whole genomes. Our study appears to be the third investigation of aphid-associated fungi against a comprehensive database of ITS sequences. While it leaves many unanswered questions, it lays the groundwork for functional investigation of the fungal microbiota in greenbug or honeydew. Its findings will guide further research that might impact future strategies to control this damaging pest.

## Figures and Tables

**Table 1:** Genera with bimodal distribution of relative abundance. Percentages refer to samples for each genus with each biotype. High samples had at least 5% of the listed genus among all counts.

Genus	% High in Biotype E	% High in Biotype K	χ^2^, *p*
*Mortierella*	0.0	14.7	3 × 10^−6^
Mortierellaceae	0.0	12.2	2 × 10^−5^
*Simplicillium*	0.0	11.5	3 × 10^−5^
Protista genus incertae sedis	0.0	11.5	3 × 10^−5^
*Fusarium*	3.9	30.1	2 × 10^−8^
*Wallemia*	0.6	6.4	0.006
Capnodiales genus incertae sedis	40.0	7.7	2 × 10^−9^
Debaryomycetaceae genus incertae sedis	21.9	9.0	0.0017
*Arthrobotrys*	12.9	3.8	0.004

**Table 2:** Module memberships of genera in biotypes E and K as revealed by WGCNA. The blue and brown modules intersect between biotypes.

Biotype	Guild Name	Dominant Taxon	Dominant Percentage	*N*	Members
E	blue	*Fusarium*	38.4	13	*Wallemia*, Hypocreales_gen_Incertae_sedis, Sordariomycetes_gen_incertae_sedis, *Fusarium*, *Chlamydosporiella*, *Thermomyces*, *Aspergillus*, *Talaromyces*, *Sarocladium*, Agaricomycetes genus incertae sedis, *Acremonium*, *Hyphopichia*, *Myrmecridium*
E	brown	*Hannaella*	41.3	10	*Microbotryomycetes*, *Fungi*, *Hannaella*, *Capnodiales_gen_Incertae_sedis*, *Arthrobotrys*, *Debaryomycetaceae_gen_incertae_sedis*, *Candida*, *Papiliotrema*, *Unassigned*, *Vexillomyces*
E	turquoise	*Pichia*	50.3	23	Mycosphaerellales, *Blastobotrys*, *Exidia*, *Curvularia*, Saccharomycetales, Agaricales, *Stemphylium*, Polyporales, *Pichia*, *Verrucocladosporium*, Athelia, *Curvibasidium*, *Wickerhamomyces*, *Chaetomidium*, *Stanjemonium*, *Rhodocollybia*, *Custingophora*, *Geotrichum*, Pleosporaceae, *Neoascochyta*, *Cephaloascus*, *Metapochonia*, *Zasmidium*
E	yellow	Unidentified Hypocreales	29.2	10	Pleosporales_gen_Incertae_sedis, Hypocreales, Agaricomycetes genus incertae sedis, Hyaloscyphaceae, *Linnemannia*, Eukaryota genus incertae sedis, *Cylindrobasidium*, *Protrudomyces*, Leotiomycetes, *Spissiomyces*
E	unaffiliated			89	
K	blue	*Wallemia*	63.3	21	Fungi genus incertae sedis, *Wallemia*, Hypocreales genus incertae sedis, Sordario*mycetes genus incertae sedis*, *Fusarium*, *Chlamydosporiella*, *Thermomyces*, *Aspergillus*, *Microascus*, *Talaromyces*, *Sarocladium*, Sordariales genus incertae sedis, Agaricomycetes, Hypocreales, Lasiosphaeriaceae gen incertae sedis, *Acremonium*, *Hyphopichia*, Helotiales genus incertae sedis, *Bipolaris*, *Myrmecridium*, *Cystofilobasidium*, *Thermothelomyces*
K	brown	*Hannaella*	40.7	28	Microbotryomycetes, *Hannaella*, Capnodiales genus incertae sedis, *Rhodotorula*, *Rigidoporus*, *Arthrobotrys*, Debaryomycetaceae genus incertae sedis, *Cystobasidium*, *Coniochaeta*, *Trichoderma*, *Candida*, *Papiliotrema*, *Hansfordia*, *Mariannaea*, Dothideomycetes, *Vexillomyces*, Agaricales, *Stemphylium*, *Rhizomucor*, *Phialemonium*, Tympanidaceae genus incertae sedis, *Neosetophoma*, Clavulinaceae genus incertae sedis, *Ceratobasidium*, Peniophoraceae, Lycoperdaceae, Coniochaetaceae, *Myriococcum*, *Mrakia*, *Candolleomyces*
K	orange	*Mortierella +* Mortierellaceae	84.0	19	Unassigned, *Neofavolus*, *Dioszegia*, *Ramularia*, *Linnemannia*, Pseudeurotiaceae genus incertae sedis, *Symmetrospora*, Cercozoa genus incertae sedis, *Rhodosporidiobolus*, Saccharomycopsidaceae genus incertae sedis, *Amyloporia*, “Branch03” genus incertae sedis, *Flammulina*, *Stanjemonium*, *Typhula*, Dictyosporiaceae genus Incertae sedis, *Meripilus*, *Agaricus*, *Mortierella* + Mortierellaceae
K	purple	*Trametes*	42.0	16	Cephalothecaceae genus incertae sedis, *Ganoderma*, *Trametes*, Pleosporales genus incertae sedis, *Hyphodontia*, *Stereum*, *Tremella*, *Trichosporon*, *Phlebia*, Xylariales genus incertae sedis, *Schizophyllum*, *Stachybotrys*, Sordariales, *Neoascochyta*, *Arthrocatena*, *Leptospora*
K	green	*Niesslia*	30.1	15	*Cercospora*, *Niesslia*, Peniophoraceae genus incertae sedis, *Bullera*, *Nigrocephalum*, *Hypoxylon*, *Steccherinum*, *Acrostalagmus*, *Chaetomidium*, *Verticillium*, *Apiotrichum*, *Kockovaella*, *Fuscoporia*, *Fibricium*, *Genolevuria*
K	red	*Periconia*	32.3	12	Agaricales genus incertae sedis, *Neodevriesia*, Dothideomycetes genus incertae sedis, *Xeromyces*, *Butlerelfia*, Polyporales, *Rhexoacrodictys*, *Phlebiopsis*, *Periconia*, *Hyphoderma*, *Pallidohirschioporus*, *Irpex*
K	unaffiliated			101	

**Table 3:** Significant rank correlations of genera to time as a continuous variable with biotype K regardless of host species or cultivar.

Genus	rho	*p*	BH adj *p*
*Sporothrix*	0.341	0.0000	0.0002
*Mortierella*	0.337	0.0000	0.0002
*Stemphylium*	0.224	0.0049	0.027
*Lecanicillium*	0.217	0.0064	0.034
*Sistotrema*	−0.207	0.0094	0.045
*Blastobotrys*	−0.209	0.0090	0.045
*Ceratobasidium*	−0.213	0.0075	0.038
*Sporisorium*	−0.226	0.0045	0.025
*Thermoascus*	−0.235	0.0032	0.018
*Thermothelomyces*	−0.237	0.0028	0.017
Sordariomycetes_gen_Incertae_sedis	−0.239	0.0026	0.016
*Stenocarpella*	−0.242	0.0024	0.015
*Sarocladium*	−0.242	0.0024	0.015
*Meyerozyma*	−0.245	0.0020	0.014
*Oliveonia*	−0.250	0.0016	0.011
*Tremella*	−0.257	0.0012	0.0087
*Xeromyces*	−0.261	0.0010	0.0076
Ustilaginaceae_gen_Incertae_sedis	−0.270	0.0006	0.0050
Saccharomycetales_gen_Incertae_sedis	−0.273	0.0006	0.0046
Microascales_gen_Incertae_sedis	−0.274	0.0005	0.0046
Sordariales_gen_Incertae_sedis	−0.277	0.0005	0.0041
*Tausonia*	−0.277	0.0005	0.0041
*Aspergillus*	−0.277	0.0005	0.0041
*Protrudomyces*	−0.280	0.0004	0.0041
Hyaloscyphaceae_gen_Incertae_sedis	−0.297	0.0002	0.0018
*Uncobasidium*	−0.300	0.0001	0.0016
Helotiales_gen_Incertae_sedis	−0.306	0.0001	0.0013
Lasiosphaeriaceae_gen_Incertae_sedis	−0.308	0.0001	0.0012
*Chlamydosporiella*	−0.320	0.0000	0.0006
Hypocreales_gen_Incertae_sedis	−0.343	0.0000	0.0002
*Microascus*	−0.355	0.0000	0.0001
*Bipolaris*	−0.384	0.0000	0.0000
*Myrmecridium*	−0.385	0.0000	0.0000
*Wallemia*	−0.385	0.0000	0.0000
*Acremonium*	−0.397	0.0000	0.0000
*Hyphopichia*	−0.420	0.0000	0.0000
Fungi_gen_Incertae_sedis	−0.453	0.0000	0.0000
*Thermomyces*	−0.469	0.0000	0.0000
*Cystofilobasidium*	−0.486	0.0000	0.0000
*Talaromyces*	−0.489	0.0000	0.0000

**Table 4:** Results of PERMANOVA of Bray–Curtis distances, which ran separately for comparisons involving host species and host cultivars.

Comparison	Df	SumOfSqs	R^2^	F	Pr(>F)
Comparisons involving susceptibility, timepoint, host species, and biotype					
susceptibility	2	0.392	0.005	1.195	0.238
timepoint	2	1.609	0.022	4.911	≤2 × 10^−4^
host_species	4	2.281	0.032	3.480	≤2 × 10^−4^
biotype	1	11.358	0.158	69.324	≤2 × 10^−4^
susceptibility:timepoint	4	1.035	0.014	1.579	0.045
susceptibility:host_species	3	0.951	0.013	1.934	0.014
timepoint:host_species	8	1.933	0.027	1.475	0.025
susceptibility:biotype	2	0.425	0.006	1.297	0.188
timepoint:biotype	2	2.477	0.035	7.559	≤2 × 10^−4^
host_species:biotype	4	2.134	0.030	3.256	≤2 × 10^−4^
susceptibility:timepoint:host_species	6	0.851	0.012	0.865	0.692
susceptibility:timepoint:biotype	4	1.013	0.014	1.546	0.045
susceptibility:host_species:biotype	3	1.981	0.028	4.031	≤2 × 10^−4^
timepoint:host_species:biotype	8	1.981	0.028	1.511	0.019
susceptibility:timepoint:host_species:biotype	6	1.121	0.016	1.141	0.242
Residual	245	40.141	0.560	NA	NA
Total	304	71.682	1	NA	NA
Comparisons involving susceptibility, timepoint, host cultivar, and biotype					
susceptibility	2	0.392	0.005	1.256	0.201
timepoint	2	1.609	0.022	5.159	≤2 × 10^−4^
host_cultivar	14	5.493	0.077	2.516	≤2 × 10^−4^
biotype	1	11.397	0.159	73.072	≤2 × 10^−4^
susceptibility:timepoint	4	1.041	0.015	1.669	0.030
timepoint:host_cultivar	28	5.108	0.071	1.170	0.079
susceptibility:biotype	2	0.428	0.006	1.371	0.155
timepoint:biotype	2	2.472	0.034	7.924	≤2 × 10^−4^
host_cultivar:biotype	14	5.770	0.080	2.642	≤2 × 10^−4^
susceptibility:timepoint:biotype	4	1.019	0.014	1.633	0.028
timepoint:host_cultivar:biotype	28	5.293	0.074	1.212	0.044
Residual	203	31.661	0.442	NA	NA
Total	304	71.682	1	NA	NA

## Data Availability

The ITS1-ITS2 amplicon reads have been deposited at NCBI SRA under bioproject PRJNA1173725. Scripts used in preparing this report have been archived at https://github.com/cfcrane/greenbug18Smycoflora (accessed on 16 July 2026).

## Supplementary Materials

The following supporting information can be downloaded at: https://www.mdpi.com/article/10.3390/insects17101025/s1, Table S1. List of genera, counts, and fractions of total as annotated in UNITE. Table S2. List of species, counts, and percentages as annotated in UNITE.
