# Supplementary material for: The Fungal Microbiome Associated with Schizaphis graminum Biotypes E and K in Response to Host Plant Species, Cultivar, and Elapsed Time
Source: Insects. 2026 Oct 5;17(10):1025. doi: 10.3390/insects17101025 (PMC13705366; doi:10.3390/insects17101025)
Supplement: Supplementary file 1 [file insects-17-01025-s001.zip › insects-4528109-supplementary.pdf]

Supplemental Table S1. List of genera, counts, and fractions of total as annotated in UNITE.

Genus	Count	Fraction of whole
Fungi_gen_Incertae_sedis	20976716	0.214871190930967
Hannaella	14081187	0.144238088574572
Fusarium	4946104	0.0506645204591805
Mortierella	4472156	0.045809719965178
Papiliotrema	3811400	0.03904138555884
Simplicillium	2733882	0.0280040250916652
Debaryomycetaceae_gen_Incertae_sedis	2239573	0.0229406603820559
Arthrobotrys	2228147	0.0228236202205942
Protista_gen_Incertae_sedis	2052726	0.0210267269802842
Capnodiales_gen_Incertae_sedis	2004677	0.0205345457516762
Naganishia	1539324	0.0157677865834013
Rhodotorula	944147	0.00967119878554392
Wallemia	883337	0.00904830256477647
Trichoderma	828451	0.00848608776502244
Cutaneotrichosporon	787932	0.00807103872754051
Aspergillus	787356	0.00806513857586871
Chlamydosporiella	721581	0.00739138427688863
Hypocreales_gen_Incertae_sedis	692535	0.00709385683685555
Trametes	585757	0.0060000957340582
Sordariales_gen_Incertae_sedis	560720	0.0057436337593936
Trichomonascus	494386	0.00506415344516258
Thermomyces	421896	0.00432161525993922
Candida	395355	0.00404974733368714
Niesslia	391586	0.00401114026484859
Malassezia	378268	0.003874719744076
Tausonia	376273	0.00385428432292108
Acremonium	348409	0.00356886448579784
Chaetomium	331072	0.00339127606646804
Hansfordia	306258	0.00313709835191248
Sistotrema	305189	0.00312614824403548
Rhodosporidiobolus	283644	0.00290545593888115
Stanjemonium	265284	0.00271738860434258
Coniochaeta	250285	0.00256374906454171
Ascomycota_gen_Incertae_sedis	235700	0.00241435025875494
Cystofilobasidium	212525	0.00217696134383493
Chaetomidium	208777	0.00213856938469275
Vishniacozyma	198165	0.00202986728479496
Sordariomycetes_gen_Incertae_sedis	188594	0.00193182847984569
Sampaiozyma	185187	0.00189692949243976
Neoascochyta	183557	0.00188023288267408
Oligophagozyma	180226	0.00184611238750262
Helotiales_gen_Incertae_sedis	171076	0.00175238601979958
Periconia	162676	0.00166634214125252
Pleosporales_gen_Incertae_sedis	158355	0.00162208076039516
Sarocladium	147372	0.00150957838919488
Apiotrichum	140258	0.00143670741872062

Mariannaea	136979	0.00140311957613065
Ramularia	135000	0.00138284804807771
Linnemannia	134174	0.00137438706668725
Exophiala	127338	0.00130436373886015
Xeromyces	114761	0.00117553351737368
Cystobasidium	107539	0.00110155626846096
Toxicocladosporium	104073	0.00106605292524142
Stemphylium	102835	0.0010533716964746
Rigidoporus	99671	0.00102096183555521
Genolevuria	96242	0.000985837495134039
Tremella	95379	0.000976997510945217
Saccharomycopsidaceae_gen_Incertae_sedis	95162	0.000974774710749418
Sporobolomyces	94108	0.000963978252655537
Myrmecridium	81070	0.000830425861167854
Talaromyces	80864	0.000828315737479676
Geotrichum	80087	0.000820356678714073
Cephalothecaceae_gen_Incertae_sedis	79453	0.000813862414547545
Bullera	78191	0.000800935346127737
Hypoxylon	76910	0.000787813654649311
Peniophora	73678	0.000754707248046443
Penicillium	73198	0.000749790454986611
Moesziomyces	71970	0.000737211659408541
Stereum	71305	0.000730399852356899
Neodevriesia	68046	0.000697016876144416
Oliveonia	64649	0.00066222032192723
Pichia	62317	0.000638332902311547
Dothideomycetes_gen_Incertae_sedis	59358	0.000608022921761459
Branch03_gen_Incertae_sedis	58741	0.0006017027940158
Agaricomycetes_gen_Incertae_sedis	56999	0.00058385893253616
Alternaria	56914	0.000582988250431815
Peniophoraceae_gen_Incertae_sedis	56038	0.000574015103097622
Amyloporia	56027	0.00057390242659
Lasiosphaeriaceae_gen_Incertae_sedis	53712	0.000550189143395186
Arthrocatena	50066	0.000512842002778213
Dioszegia	49495	0.000506993067700788
Exidia	49471	0.000506747228047797
Cercospora	48967	0.000501584595334973
Acrostalagmus	48760	0.000499464228327921
Cordycipitaceae_gen_Incertae_sedis	46749	0.000478864914071
Betamyces	46039	0.000471592157669999
Symmetrospora	45635	0.00046745385684464
Verticillium	45593	0.000467023637451905
Athelia	44985	0.000460795699576118
Neofavolus	41506	0.000425159193211212
Hyphopichia	39398	0.00040356627702345
Schizophyllum	37789	0.000387084776954139
Curvibasidium	36835	0.000377312650747723
Hyphodontia	35299	0.000361578912956261
Curvularia	32975	0.000337773439891575

Rozellomycota_gen_Incertae_sedis	32520	0.000333112729803609
Bipolaris	29688	0.000304103650750601
Saitozyma	28670	0.000293675952136208
Hyphodermella	28525	0.000292190670899384
Steccherinum	27630	0.000283022900506572
Dictyosporiaceae_gen_Incertae_sedis	27623	0.00028295119727445
Thermothelomyces	27069	0.000277276398617894
Pseudeurotiaceae_gen_Incertae_sedis	26657	0.000273056151241538
Filobasidium	26303	0.000269430016359912
Trichosporon	24526	0.000251227638719659
Sporothrix	24160	0.000247478584011538
Hyphoderma	23960	0.000245429920236608
Cercozoa_gen_Incertae_sedis	23782	0.00024360660947692
Xylariales_gen_Incertae_sedis	23461	0.000240318504118157
Scedosporium	22734	0.000232871611296287
Stachybotrys	21254	0.000217711499361805
Aureobasidium	21073	0.000215857458645494
Phaeosphaeriaceae_gen_Incertae_sedis	20577	0.000210776772483668
Veronaea	20112	0.000206013629206955
Microascales_gen_Incertae_sedis	19908	0.000203923992156527
Phialophora	19415	0.000198874035951325
Protrudomyces	19302	0.000197716540918489
Ganoderma	18897	0.000193567996774256
Catenulostroma	17689	0.000181194067573679
Leptospora	16539	0.000169414250867832
Fuscoporia	16021	0.000164108211690763
Hyaloscyphaceae_gen_Incertae_sedis	15776	0.000161598598566474
Tremellomycetes_gen_Incertae_sedis	15712	0.000160943026158497
Phlebia	15330	0.00015703007834838
Blumeria	15146	0.000155145307675445
Acrodontium	14507	0.000148599826914544
Fibricium	13648	0.00013980081600122
Rosellinia	13249	0.000135713731770234
Rhexoacrodictys	12647	0.000129547253807695
Kockovaella	12638	0.000129455063937823
Kodamaea	12638	0.000129455063937823
Meruliaceae_gen_Incertae_sedis	12179	0.000124753380574359
Chaetomiaceae_gen_Incertae_sedis	12025	0.000123175909467663
Nigrocephalum	12005	0.00012297104309017
Chloridium	11966	0.000122571553654059
Scopuloides	11263	0.00011537050048518
Conioscypha	10622	0.000108804533086529
Zopfiella	10595	0.000108527963476914
Phialemonium	10264	0.000105137424929405
Polyporales_gen_Incertae_sedis	9894	0.000101347396945784
Cryptococcus	9748	9.98518723900856e-05
Brunneomyces	9654	9.88890004158685e-05
Coprinopsis	9447	9.6768633408816e-05
Tremellales_gen_Incertae_sedis	9167	9.39005041239141e-05

Agaricales_gen_Incertae_sedis	8891	9.10733481145108e-05
Phaeoisaria	8603	8.81232722786116e-05
Cantharellales_gen_Incertae_sedis	8119	8.31655059432812e-05
Sarocladiaceae_gen_Incertae_sedis	8059	8.25509068108022e-05
Scytalidium	7652	7.83818760288197e-05
Mortierellaceae_gen_Incertae_sedis	7185	7.35982461143583e-05
Cephalotrichum	7148	7.32192433159963e-05
Crinipellis	7049	7.22051547474059e-05
Antrodiella	7020	7.19080985000411e-05
Oxyporus	6924	7.09247398880747e-05
Saccharomycetales_gen_Incertae_sedis	6844	7.01052743781028e-05
Paecilomyces	6711	6.87429129677744e-05
Meyerozyma	6618	6.77902843124319e-05
Zasmidium	6419	6.57518638563766e-05
Clavulinaceae_gen_Incertae_sedis	6390	6.54548076090118e-05
Auricularia	6215	6.36622268059481e-05
Phlebiopsis	6169	6.31910341377142e-05
Acrogenospora	6102	6.25047317731127e-05
Metapochonia	6047	6.19413492350069e-05
Uncobasidium	6035	6.18184294085111e-05
Cladophialophora	5930	6.07428809266729e-05
Trichothecium	5650	5.7874751641771e-05
Thermoascus	5553	5.688114971093e-05
Eukaryota_gen_Incertae_sedis	5491	5.62460639407017e-05
Flammulina	5355	5.48529725737493e-05
Psathyrella	5254	5.38183973674097e-05
Metarhizium	5231	5.35828010332927e-05
Spiromastigoides	4852	4.97005831798005e-05
Meristemomyces	4786	4.90245241340736e-05
Tilletiopsis	4702	4.8164085348603e-05
Narcissea	4655	4.76826493614945e-05
Uwebraunia	4501	4.61051782547984e-05
Lambertella	4432	4.53983892524476e-05
Cystostereaceae_gen_Incertae_sedis	4242	4.34521586662642e-05
Piskurozyma	4120	4.22024737635569e-05
Waitea	4098	4.19771207483146e-05
Pallidohirschioporus	4026	4.12396017893398e-05
Sporidiobolaceae_gen_Incertae_sedis	3839	3.93241011597803e-05
Parascedosporium	3839	3.93241011597803e-05
Phaeotremella	3763	3.85456089253069e-05
Cyberlindnera	3715	3.80539296193237e-05
Ophiostoma	3693	3.78285766040815e-05
Atrocalyx	3680	3.7695413458711e-05
Spissiomyces	3657	3.74598171245941e-05
Umbelopsis	3657	3.74598171245941e-05
Kiflimonium	3628	3.71627608772292e-05
Leucosporidium	3615	3.70295977318588e-05
Custingophora	3549	3.63535386861319e-05
Typhula	3293	3.37312490542216e-05

Fibrodontia	3270	3.34956527201046e-05
Beauveria	3214	3.29220268631242e-05
Punctularia	3149	3.2256211136272e-05
Tyromyces	3019	3.09245796825675e-05
Amphobotrys	3016	3.08938497259436e-05
Rasamsonia	3006	3.07914165371971e-05
Mycoacia	2952	3.0238277317966e-05
Zalaria	2902	2.97261113742335e-05
Phaeosphaeria	2766	2.83330200072812e-05
Mycothermus	2713	2.77901241069247e-05
Gloeophyllum	2694	2.75955010483064e-05
Vanakripa	2672	2.73701480330641e-05
Fusicolla	2571	2.63355728267245e-05
Microascus	2535	2.59668133472371e-05
Irpex	2516	2.57721902886187e-05
Ophiocordyceps	2480	2.54034308091313e-05
Sporisorium	2268	2.32318472077056e-05
Ustilaginaceae_gen_Incertae_sedis	2185	2.23816517411097e-05
Mycoarthris	2159	2.21153254503688e-05
Cercophora	2082	2.13265898970207e-05
Bjerkandera	2068	2.11831834327756e-05
Conocybe	2065	2.11524534761517e-05
Cytospora	1998	2.04661511115502e-05
Meripilus	1890	1.9359872673088e-05
Neosetophoma	1799	1.84277306554949e-05
Chytridiomycota_gen_Incertae_sedis	1790	1.8335540785623e-05
Blastobotrys	1778	1.82126209591272e-05
Coniophora	1710	1.7516075275651e-05
Tetrapyrgos	1624	1.66351498524312e-05
Crustomyces	1614	1.65327166636847e-05
Wickerhamomyces	1465	1.50064621513619e-05
Subulicystidium	1451	1.48630556871168e-05
Byssomerulius	1448	1.48323257304928e-05
Robbauera	1432	1.46684326284984e-05
Sebacinales_gen_Incertae_sedis	1431	1.46581893096238e-05
Resupinatus	1420	1.45455128020026e-05
Cladosporium	1413	1.44738095698801e-05
Fusariella	1413	1.44738095698801e-05
Diaporthe	1309	1.34085044069165e-05
Thyridium	1265	1.29577983764319e-05
Cyphellaceae_gen_Incertae_sedis	1239	1.2691472085691e-05
Phanerodontia	1220	1.24968490270727e-05
Baeospora	1191	1.21997927797078e-05
Microdochium	1140	1.16773835171007e-05
Neoerysiphe	1139	1.1667140198226e-05
Hohenbuehelia	1125	1.15237337339809e-05
Diatrypaceae_gen_Incertae_sedis	1098	1.12471641243654e-05
Hyalorbilia	1081	1.10730277034964e-05
Lentinellus	1077	1.10320544279978e-05

Paraphaeosphaeria	1040	1.06530516296357e-05
Serendipitaceae_gen_Incertae_sedis	1023	1.04789152087667e-05
Rhizopus	983	1.00691824537807e-05
Clitopilus	952	9.75163956866654e-06
Mrakia	930	9.52628655342425e-06
Ciboria	919	9.4136100458031e-06
Bionectriaceae_gen_Incertae_sedis	911	9.33166349480591e-06
Ustilago	894	9.15752707393686e-06
Sterigmatomyces	889	9.10631047956361e-06
Vararia	871	8.92193073981992e-06
Lecanicillium	862	8.82974086994807e-06
Steccherinaceae_gen_Incertae_sedis	857	8.77852427557482e-06
Armillaria	857	8.77852427557482e-06
Monacrosporium	852	8.72730768120157e-06
Coprinellus	827	8.47122470933533e-06
Xenasmatella	804	8.23562837521838e-06
Mucor	796	8.15368182422119e-06
Ascotaiwania	795	8.14343850534654e-06
Kondoa	770	7.88735553348029e-06
Hapalopilus	767	7.85662557685634e-06
Leptoxyphium	749	7.67224583711265e-06
Cylindrobasidium	698	7.14983657450551e-06
Hymenochaetaceae_gen_Incertae_sedis	697	7.13959325563086e-06
Neurospora	696	7.12934993675621e-06
Apioperdon	676	6.92448355926322e-06
Xanthocarpia	672	6.88351028376462e-06
Pleosporaceae_gen_Incertae_sedis	656	6.71961718177022e-06
Ramichloridium	640	6.55572407977583e-06
Cephaloascus	636	6.51475080427723e-06
Phellinus	631	6.46353420990398e-06
Golubevia	622	6.37134434003213e-06
Entyloma	573	5.8694217151743e-06
Oidiodendron	556	5.69528529430525e-06
Xylodon	552	5.65431201880665e-06
Acrophialophora	540	5.53139219231085e-06
Microcyclospora	538	5.51090555456156e-06
Cyphellophora	517	5.29579585819391e-06
Butlerelfia	492	5.03971288632767e-06
Dacryobolus	488	4.99873961082907e-06
Phaeococcomyces	486	4.97825297307977e-06
Taphrina	449	4.59925017471773e-06
Gliomastix	447	4.57876353696843e-06
Microbotryum	436	4.46608702934728e-06
Eichleriella	434	4.44560039159798e-06
Mutinus	413	4.23049069523034e-06
Debaryomyces	401	4.10757086873454e-06
Peniophorella	397	4.06659759323594e-06
Botryosphaeriales_gen_Incertae_sedis	394	4.03586763661199e-06
Westerdykella	391	4.00513767998804e-06

Ceratobasidium	387	3.96416440448945e-06
Basidiomycota_gen_Incertae_sedis	383	3.92319112899085e-06
Rhodocollybia	383	3.92319112899085e-06
Sphaerobolus	377	3.86173121574295e-06
Colletotrichum	372	3.8105146213697e-06
Myriococcum	366	3.7490547081218e-06
Tympanidaceae_gen_Incertae_sedis	364	3.7285680703725e-06
Bolbitius	357	3.65686483824995e-06
Cadophora	340	3.48272841738091e-06
Lithohypha	325	3.32907863426116e-06
Anthracocystis	320	3.27786203988791e-06
Vexillomyces	314	3.21640212664002e-06
Clonostachys	313	3.20615880776537e-06
Purpureocillium	305	3.12421225676817e-06
Sakaguchia	299	3.06275234352027e-06
Coniosporium	295	3.02177906802167e-06
Sistotremastrum	294	3.01153574914702e-06
Burgoa	294	3.01153574914702e-06
Candolleomyces	293	3.00129243027237e-06
Erythrobasidium	289	2.96031915477377e-06
Psathyrellaceae_gen_Incertae_sedis	279	2.85788596602727e-06
Coniocessia	277	2.83739932827798e-06
Idriella	276	2.82715600940333e-06
Pyxidiophora	276	2.82715600940333e-06
Skeletocutis	275	2.81691269052868e-06
Gymnopus	262	2.68374954515823e-06
Myriangium	240	2.45839652991594e-06
Bensingtonia	237	2.42766657329199e-06
Ascotricha	236	2.41742325441734e-06
Radulomyces	228	2.33547670342014e-06
Galerina	226	2.31499006567084e-06
Allocucurbitaria	211	2.16134028255109e-06
Entoloma	206	2.11012368817784e-06
Trametopsis	205	2.09988036930319e-06
Melanoporia	197	2.017933818306e-06
Yamadazyma	197	2.017933818306e-06
Trichomerium	195	1.9974471805567e-06
Sebacinaceae_gen_Incertae_sedis	192	1.96671722393275e-06
Itersonilia	190	1.94623058618345e-06
Pleurotus	188	1.92574394843415e-06
Lyomyces	186	1.90525731068485e-06
Eidernor	179	1.8335540785623e-06
Agaricus	164	1.67990429544256e-06
Stenocarpella	163	1.66966097656791e-06
Ceriporia	157	1.60820106332001e-06
Fomitiporia	155	1.58771442557071e-06
Thelebolaceae_gen_Incertae_sedis	155	1.58771442557071e-06
Sidera	155	1.58771442557071e-06
Didymocyrtis	149	1.52625451232281e-06

Torula	145	1.48528123682421e-06
Rhizochaete	145	1.48528123682421e-06
Rhizomucor	142	1.45455128020026e-06
Ophiognomonia	135	1.38284804807771e-06
Pithoascus	121	1.23944158383262e-06
Parasola	119	1.21895494608332e-06
Knufia	117	1.19846830833402e-06
Actinomortierella	112	1.14725171396077e-06
Dentocorticium	111	1.13700839508612e-06
Rhizophlyctis	106	1.08579180071287e-06
Parastagonospora	104	1.06530516296357e-06
Verrucocladosporium	101	1.03457520633962e-06
Efibula	99	1.01408856859032e-06
Neovaginatispora	97	9.93601930841024e-07
Crepidotus	93	9.52628655342425e-07
Chaetosphaeronema	92	9.42385336467775e-07
Neopyrenochaeta	86	8.80925423219877e-07
Dissoconium	83	8.50195466595928e-07
Ceratobasidiaceae_gen_Incertae_sedis	78	7.98978872222679e-07
Inopinatum	77	7.88735553348029e-07
Artomyces	72	7.37518958974781e-07
Hydnoporia	70	7.17032321225481e-07
Kurtzmaniella	65	6.65815726852232e-07
Parathyridaria	63	6.45329089102933e-07
Pyricularia	63	6.45329089102933e-07
Sphagnurus	60	6.14599132478984e-07
Phyllozyma	59	6.04355813604334e-07
Xylobolus	57	5.83869175855035e-07
Dothiora	57	5.83869175855035e-07
Lecanoromycetes_gen_Incertae_sedis	53	5.42895900356436e-07
Lepista	52	5.32652581481786e-07
Mortierellales_gen_Incertae_sedis	51	5.22409262607136e-07
Myxomphalia	45	4.60949349359238e-07
Spizellomycetales_gen_Incertae_sedis	45	4.60949349359238e-07
Graphostromataceae_gen_Incertae_sedis	41	4.19976073860639e-07
Zygoascus	38	3.8924611723669e-07
Ascobolus	38	3.8924611723669e-07
Mycena	37	3.7900279836204e-07
Volvariella	34	3.48272841738091e-07
Cystoderma	31	3.17542885114142e-07
Ossicaulis	31	3.17542885114142e-07
Papulaspora	30	3.07299566239492e-07
Laetisaria	28	2.86812928490192e-07
Strobilurus	27	2.76569609615543e-07
Udeniomyces	26	2.66326290740893e-07
Neptunomyces	24	2.45839652991594e-07
Ustilaginales_gen_Incertae_sedis	21	2.15109696367644e-07
Berkeleyomyces	21	2.15109696367644e-07
Orbiliaceae_gen_Incertae_sedis	20	2.04866377492995e-07

Thermochaetoides	19	1.94623058618345e-07
Allocryptovalsa	19	1.94623058618345e-07
Pseudozyma	18	1.84379739743695e-07
Collybiopsis	16	1.63893101994396e-07
Nectriaceae_gen_Incertae_sedis	16	1.63893101994396e-07
Actinomucor	14	1.43406464245096e-07
Phaeoacremonium	14	1.43406464245096e-07
Phanerochaete	13	1.33163145370447e-07
Polyschema	13	1.33163145370447e-07
Chaetosphaeria	12	1.22919826495797e-07
Panellus	8	8.19465509971979e-08
Gloeocystidiellum	8	8.19465509971979e-08
Saccharomyces	8	8.19465509971979e-08
Iodophanus	7	7.17032321225481e-08
Rhinocladiella	6	6.14599132478984e-08
Neocucurbitaria	6	6.14599132478984e-08
Scheffersomyces	5	5.12165943732487e-08
Meira	5	5.12165943732487e-08

There were 479 total counts summing to 97624609.

Supplemental Table S2. List of species, counts, and percentages as annotated in UNITE.

Species	Count	Fraction of whole
Fungi_sp	20976716	0.214871190930967
Hannaella_luteola	9087870	0.0930899503013631
Fusarium_nygamai	4694269	0.0480848942503831
Mortierella_sp	4448214	0.0455644744246812
Papiliotrema_mangalensis	3182296	0.0325972726815223
Simplicillium_aogashimaense	2733882	0.0280040250916652
Hannaella_zeae	2567127	0.0262959004527229
Hannaella_sinensis	2377043	0.024348809427754
Debaryomycetaceae_sp	2239573	0.0229406603820559
Arthrobotrys_amerosporus	2228071	0.0228228417283597
Protista_sp	2052726	0.0210267269802842
Capnodiales_sp	2004677	0.0205345457516762
Naganishia_albida	1537491	0.0157490105799041
Rhodotorula_toruloides	905748	0.00927786558407624
Trichoderma_sp	828336	0.00848490978335186
Chlamydosporiella_restricta	721581	0.00739138427688863
Hypocreales_sp	692535	0.00709385683685555
Aspergillus_versicolor	576384	0.00590408510624611
Sordariales_sp	560720	0.0057436337593936
Cutaneotrichosporon_moniliiforme	549806	0.00563183817719567
Trametes_versicolor	503857	0.00516116791822439
Trichomonascus_vanleenenianus	494386	0.00506415344516258
Thermomyces_dupontii	403383	0.00413198069761283
Tausonia_pullulans	376273	0.00385428432292108

Niesslia_indica	366451	0.00375367444493427
Chaetomium_ovatoascomatis	330019	0.00338048985169303
Hansfordia_pruni	298344	0.0030560327263385
Malassezia_restricta	276422	0.00283147868996843
Stanjemonium_dichromosporum	265284	0.00271738860434258
Rhodosporidiobolus_colostri	253515	0.00259683498450683
Ascomycota_sp	235700	0.00241435025875494
Coniochaeta_fasciculata	218089	0.00223395517005349
Candida_maltosa	209328	0.00214421345339268
Chaetomidium_sp	208777	0.00213856938469275
Fusarium_poae	205523	0.00210523762507464
Sordariomycetes_sp	188594	0.00193182847984569
Candida_sake	185659	0.00190176433894859
Sampaiozyma_sp	185187	0.00189692949243976
Oligophagozyma_castellii	180226	0.00184611238750262
Cystofilobasidium_infirmominiatum	179784	0.00184158484056003
Helotiales_sp	171076	0.00175238601979958
Periconia_celtidis	162410	0.00166361741843186
Pleosporales_sp	158355	0.00162208076039516
Wallemia_sebi	154853	0.00158620865769613
Acremonium_sclerotigenum	151031	0.00154705869295722
Acremonium_fusidioides	142022	0.0014547766332155
Apiotrichum_scarabaeorum	138920	0.00142300185806634
Mariannaea_camelliae	136757	0.00140084555934047
Linnemannia_hyalina	134174	0.00137438706668725
Sistotrema_porulosum	127759	0.00130867617610638
Xeromyces_bisporus	114761	0.00117553351737368
Toxicocladosporium_strelitziae	104000	0.00106530516296357
Stemphylium_vesicarium	102835	0.0010533716964746
Papiliotrema_flavescens	102338	0.0010482807669939
Rigidoporus_pouzarii	99671	0.00102096183555521
Genolevuria_amylolytica	96242	0.000985837495134039
Tremella_sp	95379	0.000976997510945217
Saccharomycopsidaceae_sp	95162	0.000974774710749418
Cystobasidium_benthicum	88076	0.00090219055320365
Sarocladium_implicatum	83631	0.000856659000805832
Cephalothecaceae_sp	79453	0.000813862414547545
Geotrichum_pandrosioniae	78280	0.000801847001507581
Hypoxylon_lenormandii	74591	0.000764059398178998
Moesziomyces_parantarcticus	71970	0.000737211659408541
Sistotrema_brinkmannii	71900	0.000736494627087316
Exophiala_sp	70459	0.000721734004588945
Neodevriesia_scadoxi	67967	0.000696207653953318
Vishniacozyma_victoriae	67421	0.00069061480184776
Oliveonia_sp	64649	0.00066222032192723
Stereum_complicatum	63755	0.000653062794853294
Malassezia_globosa	63754	0.000653052551534419
Sistotrema_sp	62870	0.000643997457649229
Dothideomycetes_sp	59358	0.000608022921761459

Branch03_sp	58741	0.0006017027940158
Peniophora_albobadia	58355	0.000597748872930185
Agaricomycetes_sp	56999	0.00058385893253616
Peniophoraceae_sp	56038	0.000574015103097622
Amyloporia_xantha	56027	0.00057390242659
Alternaria_sp	55521	0.000568719307239428
Pichia_fermentans	55405	0.000567531082249968
Lasiosphaeriaceae_sp	53712	0.000550189143395186
Aspergillus_lucknowensis	53147	0.000544401668231009
Arthrocatena_tenebrosa	50066	0.000512842002778213
Exidia_pithya	49471	0.000506747228047797
Cercospora_coniogrammes	48967	0.000501584595334973
Acrostalagmus_luteoalbus	48760	0.000499464228327921
Cordycipitaceae_sp	46749	0.000478864914071
Betamyces_sp	46039	0.000471592157669999
Verticillium_dahliae	45593	0.000467023637451905
Aspergillus_flavus	41803	0.000428201458916983
Neofavolus_sp	39496	0.000404570122273166
Talaromyces_sp	38924	0.000398710943876866
Dioszegia_zsoltii	37881	0.000388027162290606
Schizophyllum_umbrinum	37789	0.000387084776954139
Curvibasidium_cygneicollum	36835	0.000377312650747723
Sarocladium_ochraceum	36727	0.000376206372309261
Athelia_arachnoidea	35757	0.00036627035300085
Malassezia_sp	35498	0.000363617333412316
Hyphodontia_arguta	35299	0.000361578912956261
Rhodotorula_diobovata	34458	0.00035296428178268
Hyphopichia_burtonii	33615	0.000344329163971351
Rozellomycota_sp	32520	0.000333112729803609
Curvularia_inaequalis	31820	0.000325942406591354
Cystofilobasidium_capitatum	31046	0.000318014077782376
Saitozyma_podzolica	28670	0.000293675952136208
Bipolaris_microstegii	27884	0.000285624703500733
Dictyosporiaceae_sp	27623	0.00028295119727445
Hyphodermella_rosae	27105	0.000277645158097381
Thermothelomyces_myriococcoides	26843	0.000274961408552223
Symmetrospora_oryzicola	26788	0.000274398026014117
Trametes_membranacea	26770	0.000274213646274373
Pseudeurotiaceae_sp	26657	0.000273056151241538
Acremonium_charticola	26020	0.000266531157118386
Sistotrema_sernanderi	25994	0.000266264830827645
Sporobolomyces_roseus	24924	0.00025530447963177
Hyphoderma_setigerum	23960	0.000245429920236608
Cercozoa_sp	23782	0.00024360660947692
Acremonium_sp	23749	0.000243268579954056
Xylariales_sp	23461	0.000240318504118157
Niesslia_loricata	22762	0.000233158424224777
Sarocladium_agarici	22362	0.000229061096674917
Exophiala_attenuata	21679	0.000222064909883532

Rhodosporidiobolus_sp	21572	0.000220968874763944
Stachybotrys_sp	21254	0.000217711499361805
Aureobasidium_pullulans	21073	0.000215857458645494
Phaeosphaeriaceae_sp	20577	0.000210776772483668
Veronaea_botryosa	19931	0.000204159588490644
Microascales_sp	19908	0.000203923992156527
Trichosporon_ovoides	19524	0.000199990557708661
Protrudomyces_lateralis	19302	0.000197716540918489
Phialophora_cyclaminis	18294	0.000187391275492842
Catenulostroma_sp	17689	0.000181194067573679
Sporobolomyces_carnicolor	17490	0.000179155647117624
Steccherinum_tenue	17210	0.000176287517832722
Aspergillus_fumigatus	16521	0.000169229871128088
Sistotrema_raduloides	15863	0.000162489767308569
Fuscoporia_ferruginosa	15855	0.000162407820757571
Hyaloscyphaceae_sp	15776	0.000161598598566474
Tremellomycetes_sp	15712	0.000160943026158497
Aspergillus_caespitosus	15405	0.000157798327263979
Phlebia_tremellosa	15303	0.000156753508738765
Blumeria_americana	15146	0.000155145307675445
Acrodontium_pigmentosum	14500	0.000148528123682421
Aspergillus_terreus	14319	0.000146674082966109
Fibricium_sp	13648	0.00013980081600122
Vishniacozyma_tephrensis	13497	0.000138254074851147
Cystobasidium_minutum	13437	0.000137639475718668
Rosellinia_subiculata	13249	0.000135713731770234
Talaromyces_piceae	12967	0.000132825115847583
Aspergillus_deflectus	12775	0.00013085839862365
Papiliotrema_laurentii	12749	0.000130592072332909
Rhexoacrodictys_sp	12647	0.000129547253807695
Kodamaea_ohmeri	12638	0.000129455063937823
Meruliaceae_sp	12179	0.000124753380574359
Chaetomiaceae_sp	12025	0.000123175909467663
Nigrocephalum_collariferum	12005	0.00012297104309017
Chloridium_humicola	11966	0.000122571553654059
Talaromyces_columbinus	11465	0.000117439650897859
Scopuloides_sp	11263	0.00011537050048518
Conioscypha_pleiomorpha	10622	0.000108804533086529
Zopfiella_sp	10595	0.000108527963476914
Steccherinum_ochraceum	10399	0.000106520272977483
Polyporales_sp	9894	0.000101347396945784
Cryptococcus_watticus	9748	9.98518723900856e-05
Aspergillus_insuetus	9723	9.95957894182193e-05
Dioszegia_sp	9671	9.90631368367375e-05
Tremellales_sp	9167	9.39005041239141e-05
Vishniacozyma_globispora	9137	9.35932045576746e-05
Coniochaeta_ligniaria	8942	9.15957573771179e-05
Phialemonium_inflatum	8906	9.12269978976305e-05
Agaricales_sp	8891	9.10733481145108e-05

Rhodosporidiobolus_oreadorum	8432	8.63716647510465e-05
Cantharellales_sp	8119	8.31655059432812e-05
Sarocladiaceae_sp	8059	8.25509068108022e-05
Penicillium_oxalicum	8056	8.25201768541782e-05
Stereum_subtomentosum	7525	7.70809745317392e-05
Mortierellaceae_sp	7185	7.35982461143583e-05
Crinipellis_zonata	7049	7.22051547474059e-05
Antrodiella_onychoides	7020	7.19080985000411e-05
Oxyporus_piceicola	6924	7.09247398880747e-05
Trametes_cubensis	6920	7.08837666125761e-05
Saccharomycetales_sp	6844	7.01052743781028e-05
Scytalidium_circinatum	6840	7.00643011026042e-05
Phaeoisaria_sp	6504	6.66225459607218e-05
Clavulinaceae_sp	6390	6.54548076090118e-05
Auricularia_scissa	6215	6.36622268059481e-05
Acrogenospora_carmichaeliana	6102	6.25047317731127e-05
Uncobasidium_sp	6035	6.18184294085111e-05
Cladophialophora_minourae	5930	6.07428809266729e-05
Hyphopichia_pseudoburtonii	5783	5.92371130520994e-05
Trichothecium_roseum	5650	5.7874751641771e-05
Thermoascus_crustaceus	5553	5.688114971093e-05
Paecilomyces_maximus	5520	5.65431201880665e-05
Eukaryota_sp	5491	5.62460639407017e-05
Flammulina_velutipes	5355	5.48529725737493e-05
Exophiala_equina	5099	5.2230682941839e-05
Trichosporon_sp	5002	5.1237081010998e-05
Metarhizium_sp	4894	5.01308025725358e-05
Spiromastigoides_warcupii	4852	4.97005831798005e-05
Meristemomyces_frigidus	4786	4.90245241340736e-05
Penicillium_citrinum	4779	4.89528209019511e-05
Tilletiopsis_washingtonensis	4702	4.8164085348603e-05
Narcissea_lahorensis	4655	4.76826493614945e-05
Metapochonia_sp	4529	4.63919911832886e-05
Uwebraunia_musae	4501	4.61051782547984e-05
Lambertella_hicoriae	4432	4.53983892524476e-05
Coprinopsis_urticicola	4403	4.51013330050828e-05
Sarocladium_glaucum	4330	4.43535707272333e-05
Phlebiopsis_amethystea	4297	4.40155412043699e-05
Peniophora_simulans	4287	4.39131080156234e-05
Cephalotrichum_columnare	4277	4.38106748268769e-05
Cystostereaceae_sp	4242	4.34521586662642e-05
Pallidohirschioporus_subchartaceus	4026	4.12396017893398e-05
Coprinopsis_pachyderma	3902	3.99694302488832e-05
Acremonium_nigrosclerotium	3887	3.98157804657635e-05
Parascedosporium_putredinis	3839	3.93241011597803e-05
Sporidiobolaceae_sp	3839	3.93241011597803e-05
Exophiala_mesophila	3788	3.88016918971732e-05
Pichia_kudriavzevii	3787	3.87914485782985e-05
Phaeotremella_sp	3763	3.85456089253069e-05

Atrocalyx_bambusae	3680	3.7695413458711e-05
Spissiomyces_sp	3657	3.74598171245941e-05
Kiflimonium_sp	3628	3.71627608772292e-05
Ophiostoma_tetropii	3614	3.70193544129841e-05
Leucosporidium_muscorum	3609	3.69681378186109e-05
Ganoderma_lobatum	3601	3.68861912676137e-05
Custingophora_olivacea	3549	3.63535386861319e-05
Umbelopsis_isabellina	3535	3.62101322218868e-05
Fusarium_sp	3479	3.56365063649064e-05
Papiliotrema_fusca	3423	3.5062880507926e-05
Brunneomyces_brunnescens	3371	3.45302279264442e-05
Symmetrospora_suhii	3232	3.31064066028679e-05
Peniophora_sp	3164	3.24098609193917e-05
Punctularia_sp	3149	3.2256211136272e-05
Tyromyces_galactinus	3019	3.09245796825675e-05
Amphobotrys_ricini	3016	3.08938497259436e-05
Cyberlindnera_jadinii	3016	3.08938497259436e-05
Rasamsonia_emersonii	3006	3.07914165371971e-05
Aspergillus_crustosus	2988	3.06070367974534e-05
Zalaria_alba	2902	2.97261113742335e-05
Piskurozyma_capsuligena	2818	2.88656725887629e-05
Mycothermus_thermophilus	2713	2.77901241069247e-05
Gloeophyllum_trabeum	2694	2.75955010483064e-05
Pichia_norvegensis	2677	2.74213646274373e-05
Vanakripa_minutiellipsoidea	2672	2.73701480330641e-05
Typhula_sp	2613	2.67657922194597e-05
Cystobasidium_psychroaquaticum	2600	2.66326290740893e-05
Malassezia_sympodialis	2594	2.65711691608414e-05
Fusicolla_ossicola	2571	2.63355728267245e-05
Filobasidium_chernovii	2488	2.54853773601285e-05
Ophiocordyceps_termiticola	2480	2.54034308091313e-05
Cystobasidium_calyptogenae	2361	2.4184475863048e-05
Cephalotrichum_asperulum	2344	2.4010339442179e-05
Hypoxylon_sp	2319	2.37542564703127e-05
Symmetrospora_foliicola	2312	2.36825532381902e-05
Waitea_circinata	2273	2.32830638020788e-05
Phaeosphaeria_lycopodina	2245	2.29962508735886e-05
Vishniacozyma_sp	2229	2.28323577715942e-05
Ustilaginaceae_sp	2185	2.23816517411097e-05
Niesslia_exilis	2168	2.22075153202406e-05
Mycoarthris_corallina	2159	2.21153254503688e-05
Phaeoisaria_clematidis	2099	2.15007263178898e-05
Mycoacia_fuscoatra	2096	2.14699963612658e-05
Cercophora_fici	2082	2.13265898970207e-05
Bjerkandera_adusta	2068	2.11831834327756e-05
Conocybe_sp	2065	2.11524534761517e-05
Cytospora_sp	1948	1.99539851678177e-05
Meripilus_sumstinei	1890	1.9359872673088e-05
Phlebiopsis_gigantea	1872	1.91754929333443e-05

Waitea_sp	1825	1.86940569462358e-05
Geotrichum_sp	1807	1.85096772064921e-05
Chytridiomycota_sp	1790	1.8335540785623e-05
Exophiala_alcalophila	1714	1.75570485511496e-05
Coniophora_marmorata	1710	1.7516075275651e-05
Cystofilobasidium_macerans	1695	1.73624254925313e-05
Talaromyces_rugulosus	1649	1.68912328242974e-05
Tetrapyrgos_nigripes	1624	1.66351498524312e-05
Crustomyces_subabruptus	1614	1.65327166636847e-05
Aspergillus_clavatophorus	1594	1.63278502861917e-05
Neosetophoma_poaceicola	1594	1.63278502861917e-05
Penicillium_decumbens	1554	1.59181175312057e-05
Vishniacozyma_psychrotolerans	1540	1.57747110669606e-05
Metapochonia_bulbillosa	1518	1.55493580517183e-05
Wickerhamomyces_anomalus	1465	1.50064621513619e-05
Subulicystidium_perlongisporum	1451	1.48630556871168e-05
Byssomerulius_corium	1448	1.48323257304928e-05
Robbauera_albescens	1432	1.46684326284984e-05
Sebacinales_sp	1431	1.46581893096238e-05
Fusariella_sp	1413	1.44738095698801e-05
Cladosporium_cladosporioides	1413	1.44738095698801e-05
Alternaria_tellustris	1372	1.40538334960194e-05
Coniochaeta_velutina	1370	1.40333468582701e-05
Phialemonium_sp	1358	1.39104270317743e-05
Acremonium_acutatum	1340	1.37260472920306e-05
Apiotrichum_chiropterorum	1338	1.37055606542813e-05
Coniochaeta_prunicola	1321	1.35314242334123e-05
Diaporthe_sp	1309	1.34085044069165e-05
Scedosporium_minutisporum	1293	1.32446113049221e-05
Resupinatus_applicatus	1288	1.31933947105489e-05
Hannaella_phetchabunensis	1286	1.31729080727996e-05
Thyridium_curvatum	1265	1.29577983764319e-05
Cyphellaceae_sp	1239	1.2691472085691e-05
Phanerodontia_chrysosporium	1220	1.24968490270727e-05
Sporisorium_graminicola	1216	1.24558757515741e-05
Aspergillus_pernambucoensis	1177	1.20563863154627e-05
Microdochium_sp	1140	1.16773835171007e-05
Neoerysiphe_maquii	1139	1.1667140198226e-05
Fusarium_solani	1138	1.16568968793514e-05
Phialophora_chinensis	1121	1.14827604584823e-05
Hohenbuehelia_petaloides	1104	1.13086240376133e-05
Diatrypaceae_sp	1098	1.12471641243654e-05
Hyalorbilia_fusarina	1081	1.10730277034964e-05
Lentinellus_subaustralis	1077	1.10320544279978e-05
Penicillium_implicatum	1062	1.0878404644878e-05
Sporisorium_sp	1052	1.07759714561315e-05
Blastobotrys_attinorum	1046	1.07145115428836e-05
Paraphaeosphaeria_michotii	1040	1.06530516296357e-05
Serendipitaceae_sp	1023	1.04789152087667e-05

Neoascochyta_sp	1012	1.03662387011455e-05
Rhizopus_arrhizus	983	1.00691824537807e-05
Papiliotrema_sp	927	9.4955565968003e-06
Bionectriaceae_sp	911	9.33166349480591e-06
Scedosporium_sp	892	9.13704043618756e-06
Sterigmatomyces_halophilus	889	9.10631047956361e-06
Coniochaeta_pulveracea	880	9.01412060969176e-06
Vararia_sp	871	8.92193073981992e-06
Lecanicillium_psalliotae	862	8.82974086994807e-06
Steccherinaceae_sp	857	8.77852427557482e-06
Armillaria_borealis	857	8.77852427557482e-06
Mycoacia_uda	856	8.76828095670017e-06
Monacrosporium_doedycoides	852	8.72730768120157e-06
Baeospora_myosura	817	8.36879152058883e-06
Scytalidium_sp	812	8.31757492621558e-06
Xenasmatella_christiansenii	804	8.23562837521838e-06
Ascotaiwania_sp	795	8.14343850534654e-06
Paecilomyces_tabacinus	781	8.00003204110144e-06
Mucor_racemosus	779	7.97954540335214e-06
Aspergillus_wentii	773	7.91808549010424e-06
Hapalopilus_ribicola	757	7.75419238810985e-06
Leptoxyphium_madagascariense	749	7.67224583711265e-06
Ustilago_sp	732	7.4981094162436e-06
Filobasidium_sp	728	7.457136140745e-06
Leptospora_macarangae	726	7.4366495029957e-06
Aspergillus_piperis	713	7.30348635762526e-06
Penicillium_olsonii	700	7.17032321225481e-06
Cyberlindnera_fabianii	699	7.16007989338016e-06
Hymenochaetaceae_sp	697	7.13959325563086e-06
Symmetrospora_coprosmae	696	7.12934993675621e-06
Neurospora_sp	696	7.12934993675621e-06
Bullera_alba	685	7.01667342913507e-06
Typhula_fistulosa	680	6.96545683476182e-06
Apioperdon_sp	676	6.92448355926322e-06
Xanthocarpia_crenulatella	672	6.88351028376462e-06
Pleosporaceae_sp	656	6.71961718177022e-06
Ramichloridium_cucurbitae	640	6.55572407977583e-06
Phellinus_robiniae	631	6.46353420990398e-06
Mrakia_soli	631	6.46353420990398e-06
Coprinopsis_strossmayeri	627	6.42256093440538e-06
Golubevia_pallescens	622	6.37134434003213e-06
Cylindrobasidium_torrendii	597	6.11526136816589e-06
Meyerozyma_guilliermondii	588	6.02307149829404e-06
Entyloma_calendulae	573	5.8694217151743e-06
Penicillium_sp	567	5.8079618019264e-06
Cephaloascus_fragrans	561	5.7465018886785e-06
Kondoa_malvinella	550	5.63382538105735e-06
Acrophialophora_levis	540	5.53139219231085e-06
Cephalotrichum_stemonitis	522	5.34701245256716e-06

Wallemia_muriae	496	5.08068616182627e-06
Butlerelfia_eustacei	492	5.03971288632767e-06
Dacryobolus_sudans	488	4.99873961082907e-06
Phaeococcomyces_sp	486	4.97825297307977e-06
Xylodon_radula	481	4.92703637870652e-06
Clitopilus_baronii	454	4.65046676909098e-06
Gliomastix_tumulicola	447	4.57876353696843e-06
Microbotryum_anomalum	436	4.46608702934728e-06
Eichleriella_shearii	434	4.44560039159798e-06
Oidiodendron_sp	422	4.32268056510219e-06
Coprinellus_micaceus	421	4.31243724622754e-06
Neoascochyta_desmazieri	418	4.28170728960359e-06
Mutinus_sp	413	4.23049069523034e-06
Meyerozyma_sp	411	4.21000405748104e-06
Paecilomyces_variotii	410	4.19976073860639e-06
Coprinellus_disseminatus	406	4.15878746310779e-06
Debaryomyces_prosopidis	401	4.10757086873454e-06
Peniophorella_guttulifera	397	4.06659759323594e-06
Taphrina_inositophila	397	4.06659759323594e-06
Botryosphaeriales_sp	394	4.03586763661199e-06
Westerdykella_dispersa	391	4.00513767998804e-06
Ceratobasidium_sp	387	3.96416440448945e-06
Rhodocollybia_butyracea	383	3.92319112899085e-06
Basidiomycota_sp	383	3.92319112899085e-06
Coprinopsis_scobicola	381	3.90270449124155e-06
Sphaerobolus_sp	377	3.86173121574295e-06
Dioszegia_hungarica	376	3.8514878968683e-06
Candida_parapsilosis	368	3.7695413458711e-06
Blastobotrys_adeninivorans	367	3.75929802699645e-06
Myriococcum_sp	366	3.7490547081218e-06
Colletotrichum_graminicola	366	3.7490547081218e-06
Blastobotrys_persicus	365	3.73881138924715e-06
Talaromyces_siglerae	365	3.73881138924715e-06
Tympanidaceae_sp	364	3.7285680703725e-06
Penicillium_brasilianum	360	3.6875947948739e-06
Bolbitius_titubans	357	3.65686483824995e-06
Rhodotorula_taiwanensis	344	3.52370169287951e-06
Cadophora_sp	340	3.48272841738091e-06
Cyphellophora_sp	339	3.47248509850626e-06
Metarhizium_rileyi	337	3.45199846075696e-06
Talaromyces_infraolivaceus	335	3.43151182300766e-06
Aspergillus_purpureus	333	3.41102518525836e-06
Cutaneotrichosporon_mucoides	326	3.33932195313581e-06
Lithohypha_sp	325	3.32907863426116e-06
Penicillium_tealii	322	3.29834867763721e-06
Sarocladium_dejongiae	322	3.29834867763721e-06
Clitopilus_sp	320	3.27786203988791e-06
Anthracocystis_abscondita	320	3.27786203988791e-06
Clonostachys_sp	313	3.20615880776537e-06

Pichia_mandshurica	305	3.12421225676817e-06
Mrakia_frigida	299	3.06275234352027e-06
Sakaguchia_sp	299	3.06275234352027e-06
Coniosporium_sp	295	3.02177906802167e-06
Burgoa_verzuoliana	294	3.01153574914702e-06
Sistotremastrum_guttuliferum	294	3.01153574914702e-06
Candolleomyces_sp	293	3.00129243027237e-06
Erythrobasidium_sp	289	2.96031915477377e-06
Aspergillus_penicillioides	287	2.93983251702447e-06
Psathyrellaceae_sp	279	2.85788596602727e-06
Coniocessia_sp	277	2.83739932827798e-06
Pyxidiophora_arvernensis	276	2.82715600940333e-06
Idriella_sp	276	2.82715600940333e-06
Skeletocutis_semipileata	275	2.81691269052868e-06
Gymnopus_androsaceus	262	2.68374954515823e-06
Vexillomyces_xylophilus	257	2.63253295078498e-06
Sistotrema_coronilla	245	2.50961312428918e-06
Coniochaeta_sp	244	2.49936980541453e-06
Bensingtonia_sp	237	2.42766657329199e-06
Ascotricha_guamensis	236	2.41742325441734e-06
Galerina_laevis	226	2.31499006567084e-06
Mariannaea_macrochlamydospora	222	2.27401679017224e-06
Kondoa_gutianensis	220	2.25353015242294e-06
Periconia_festucae	218	2.23304351467364e-06
Allocucurbitaria_mori	211	2.16134028255109e-06
Trametopsis_abieticola	205	2.09988036930319e-06
Thermothelomyces_thermophilus	204	2.08963705042855e-06
Sporobolomyces_johnsonii	199	2.0384204560553e-06
Melanoporia_nigra	197	2.017933818306e-06
Yamadazyma_olivae	197	2.017933818306e-06
Aspergillus_ochraceus	196	2.00769049943135e-06
Sebacinaceae_sp	192	1.96671722393275e-06
Itersonilia_perplexans	190	1.94623058618345e-06
Aspergillus_sclerotialis	189	1.9359872673088e-06
Niesslia_aeruginosa	187	1.9155006295595e-06
Lyomyces_sp	186	1.90525731068485e-06
Eidernor_doerrieniae	179	1.8335540785623e-06
Cyphellophora_olivacea	178	1.82331075968765e-06
Penicillium_curticaule	173	1.7720941653144e-06
Fuscoporia_contigua	166	1.70039093319186e-06
Stenocarpella_maydis	163	1.66966097656791e-06
Ustilago_maydis	162	1.65941765769326e-06
Fomitiporia_ignea	155	1.58771442557071e-06
Sidera_vulgaris	155	1.58771442557071e-06
Thelebolaceae_sp	155	1.58771442557071e-06
Didymocyrtis_brachylaenae	149	1.52625451232281e-06
Torula_hollandica	145	1.48528123682421e-06
Rhizochaete_americana	145	1.48528123682421e-06
Rhizomucor_pusillus	142	1.45455128020026e-06

Veronaea_japonica	136	1.39309136695236e-06
Ophiognomonia_sp	135	1.38284804807771e-06
Resupinatus_kavinae	132	1.35211809145376e-06
Ceriporia_purpurea	131	1.34187477257911e-06
Rhodosporidiobolus_ruineniae	125	1.28041485933122e-06
Trichomerium_dioscoreae	122	1.24968490270727e-06
Pithoascus_nidicola	121	1.23944158383262e-06
Pichia_cephalocereana	120	1.22919826495797e-06
Parasola_psathyrelloides	119	1.21895494608332e-06
Exophiala_nishimurae	117	1.19846830833402e-06
Knufia_epidermidis	117	1.19846830833402e-06
Trichoderma_longibrachiatum	115	1.17798167058472e-06
Leptospora_clematidis	114	1.16773835171007e-06
Athelia_epiphylla	114	1.16773835171007e-06
Actinomortierella_wolfii	112	1.14725171396077e-06
Dentocorticium_portoricense	111	1.13700839508612e-06
Coprinopsis_sp	110	1.12676507621147e-06
Rhizophlyctis_sp	106	1.08579180071287e-06
Parastagonospora_stipae	104	1.06530516296357e-06
Verrucocladosporium_dirinae	101	1.03457520633962e-06
Efibula_gracilis	99	1.01408856859032e-06
Penicillium_chrysogenum	98	1.00384524971567e-06
Neovaginatispora_clematidis	97	9.93601930841024e-07
Crepidotus_pseudomollis	93	9.52628655342425e-07
Chaetosphaeronema_sp	92	9.42385336467775e-07
Neopyrenochaeta_acicola	86	8.80925423219877e-07
Trametes_elegans	81	8.29708828846628e-07
Penicillium_ramusculum	80	8.19465509971978e-07
Neodevriesia_agapanthi	79	8.09222191097329e-07
Ophiostoma_sparsiannulatum	79	8.09222191097329e-07
Ceratobasidiaceae_sp	78	7.98978872222679e-07
Inopinatum_lactosum	77	7.88735553348029e-07
Arthrobotrys_conoides	76	7.7849223447338e-07
Cephaloascus_albidus	75	7.6824891559873e-07
Aspergillus_teporis	74	7.5800559672408e-07
Trichomerium_flexuosum	73	7.4776227784943e-07
Microascus_cirrosus	73	7.4776227784943e-07
Artomyces_pyxidatus	72	7.37518958974781e-07
Xylodon_apacheriensis	71	7.27275640100131e-07
Hydnoporia_rimosa	70	7.17032321225481e-07
Penicillium_multicolor	66	6.76059045726882e-07
Kurtzmaniella_quercitrusa	65	6.65815726852232e-07
Parathyridaria_ellipsoidea	63	6.45329089102933e-07
Pyricularia_oryzae	63	6.45329089102933e-07
Sporobolomyces_salmonicolor	61	6.24842451353634e-07
Sphagnurus_paluster	60	6.14599132478984e-07
Agaricus_karstomyces	57	5.83869175855035e-07
Vexillomyces_sp	57	5.83869175855035e-07
Aspergillus_niger	55	5.63382538105735e-07

Lecanoromycetes_sp	53	5.42895900356436e-07
Mortierellales_sp	51	5.22409262607136e-07
Penicillium_angulare	49	5.01922624857837e-07
Periconia_verrucosa	48	4.91679305983187e-07
Talaromyces_radicus	48	4.91679305983187e-07
Myxomphalia_maura	45	4.60949349359238e-07
Spizellomycetales_sp	45	4.60949349359238e-07
Neosetophoma_sp	44	4.50706030484588e-07
Taphrina_sp	44	4.50706030484588e-07
Penicillium_sumatraense	42	4.30219392735289e-07
Graphostromataceae_sp	41	4.19976073860639e-07
Filobasidium_wieringae	40	4.09732754985989e-07
Penicillium_brevicompactum	39	3.9948943611134e-07
Ascobolus_sp	38	3.8924611723669e-07
Xylobolus_frustulatus	38	3.8924611723669e-07
Zygoascus_meyerae	38	3.8924611723669e-07
Dothiora_laureolae	35	3.58516160612741e-07
Volvariella_volvacea	34	3.48272841738091e-07
Cystoderma_sp	31	3.17542885114142e-07
Ossicaulis_lignatilis	31	3.17542885114142e-07
Phaeosphaeria_sp	30	3.07299566239492e-07
Papulaspora_funabasensis	30	3.07299566239492e-07
Laetisaria_fuciformis	28	2.86812928490192e-07
Phlebia_acerina	27	2.76569609615543e-07
Strobilurus_stephanocystis	27	2.76569609615543e-07
Udeniomyces_megalosporus	26	2.66326290740893e-07
Ceriporia_sp	26	2.66326290740893e-07
Stereum_lobatum	25	2.56082971866243e-07
Talaromyces_delawarensis	25	2.56082971866243e-07
Neptunomyces_sp	24	2.45839652991594e-07
Fusarium_oxysporum	23	2.35596334116944e-07
Thermothelomyces_fergusii	22	2.25353015242294e-07
Dothiora_infuscans	22	2.25353015242294e-07
Ustilaginales_sp	21	2.15109696367644e-07
Coniochaeta_luteorubra	21	2.15109696367644e-07
Hohenbuehelia_pseudocyphelliformis	21	2.15109696367644e-07
Berkeleyomyces_rouxiae	21	2.15109696367644e-07
Orbiliaceae_sp	20	2.04866377492995e-07
Thermochaetoides_sp	19	1.94623058618345e-07
Xylobolus_subpileatus	19	1.94623058618345e-07
Allocryptovalsa_xishuangbanica	19	1.94623058618345e-07
Athelia_neuhoffii	18	1.84379739743695e-07
Mucor_circinelloides	17	1.74136420869045e-07
Nectriaceae_sp	16	1.63893101994396e-07
Actinomucor_elegans	14	1.43406464245096e-07
Alternaria_alternata	13	1.33163145370447e-07
Phanerochaete_burtii	13	1.33163145370447e-07
Polyschema_sp	13	1.33163145370447e-07
Chaetosphaeria_sp	12	1.22919826495797e-07

Toxicocladosporium_velox	12	1.22919826495797e-07
Pseudozyma_flocculosa	11	1.12676507621147e-07
Papiliotrema_pseudoalba	10	1.02433188746497e-07
Hapalopilus_mutans	10	1.02433188746497e-07
Talaromyces_assiutensis	10	1.02433188746497e-07
Mycena_flavoalba	10	1.02433188746497e-07
Microascus_cinereus	8	8.19465509971979e-08
Saccharomyces_cerevisiae	8	8.19465509971979e-08
Taphrina_letifera	8	8.19465509971979e-08
Panellus_serotinus	8	8.19465509971979e-08
Gloeocystidiellum_kenyense	8	8.19465509971979e-08
Phaeoacremonium_subulatum	7	7.17032321225481e-08
Penicillium_bialowiezense	7	7.17032321225481e-08
Phaeoacremonium_hungaricum	7	7.17032321225481e-08
Iodophanus_testaceus	7	7.17032321225481e-08
Pseudozyma_sp	7	7.17032321225481e-08
Colletotrichum_cliviicola	6	6.14599132478984e-08
Sporothrix_resoviensis	6	6.14599132478984e-08
Leucosporidium_creatinivorum	6	6.14599132478984e-08
Rhinocladiella_sp	6	6.14599132478984e-08
Scheffersomyces_shehatae	5	5.12165943732487e-08
Myrmecridium_schulzeri	5	5.12165943732487e-08
Aspergillus_sp	5	5.12165943732487e-08
Meira_geulakonigae	5	5.12165943732487e-08

There were 740 total counts summing to 97624609.
